# Statistical mirroring: A robust method for statistical dispersion estimation

**DOI:** 10.1016/j.mex.2024.102682

**Published:** 2024-04-16

**Authors:** Kabir Bindawa Abdullahi

**Affiliations:** Department of Biology, Faculty of Natural and Applied Sciences, Umaru Musa Yar'adua University, P.M.B., 2218 Katsina, Katsina State, Nigeria

**Keywords:** Kabirian-based isomorphic optinalysis, Customization, Dispersion estimators, Monte Carlos simulation, Temperature measurement scales, Performance evaluation, Statistical analysis, Statistical Mirroring

## Abstract

This study introduces statistical mirroring as an innovative approach to statistical dispersion estimation, drawing inspiration from the Kabirian-based isomorphic optinalysis model, aimed at enhancing robustness and mitigating biases in estimation methods. Beyond scale-invariant characteristics, the proposed estimators emphasize scaloc-invariant robustness, thereby addressing a critical gap in dispersion estimation. By highlighting statistical meanic mirroring, alongside other forms of proposed statistical mirroring, the study underscores the adaptability and customization potential. Through extensive Monte Carlo simulations and real-life applications, in comparison with classical estimators, the results of the performance evaluation of the proposed estimators demonstrate robustness, efficiency, and transformations-invariance. The research offers a paradigm shift in addressing longstanding challenges in dispersion estimation, offering a new category of dispersion estimation and increased resistance to outliers. Notable limitations include selecting and evaluating the proposed statistical meanic mirroring under Gaussian and Gaussian mixture model distributions. This research paper significantly contributes to statistical methodologies, offering avenues for expanding knowledge in dispersion estimation. It recommends further exploration of proposed estimators across various statistical *mirroring* types and encourages comparative studies to establish their effectiveness, thereby advancing statistical knowledge and tools for precise data analysis.

• The proposed methodology involves preprocessing transformations, statistical mirror design, and optimization to transform a univariate set into a bivariate one, facilitating the fitting of an *isomorphic optinalysis* model.
• Estimators rely on a foundational bijective mapping of *isoreflective pairs*, deducing the probability of proximity or deviation from any defined center. This contrasts with classical estimators that utilize average or median deviations from a mean or median center.

## Graphical abstract

**Figure:**
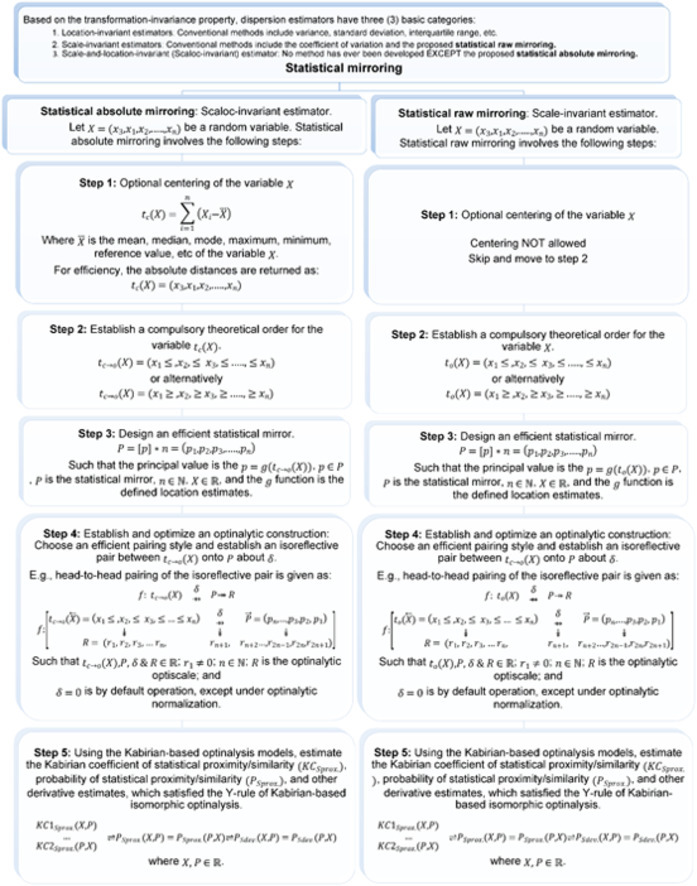

Specifications table

**Table:** 

Subject area:	Mathematics and Statistics
More specific subject area:	Data Analysis and Analytics
Name of your method:	Statistical Mirroring
Name and reference of the original method:	Kabirian-Based Isomorphic Optinalysis; K.B. Abdullahi, Kabirian-based optinalysis: A conceptually grounded framework for symmetry/asymmetry, similarity/dissimilarity, and identity/unidentity estimations in mathematical structures and biological sequences, MethodsX 11 (2023) 102,400. https://doi.org/10.1016/j.mex.2023.102400
Resource availability:	Get the Python codes/scripts and computer application for statistical mirroring and for simulating, analyzing, and evaluating dispersion estimators via these links: 1. K.B. Abdullahi, A Python Code for Statistical Mirroring, Mendeley Data V3 (2024). https://doi.org/10.17632/ppfvc65m2v.3 (https://data.mendeley.com/datasets/ppfvc65m2v/3) 2. K.B. Abdullahi, Python Scripts for Simulating, Analyzing, and Evaluating Dispersion Estimators, Mendeley Data V2 (2024). https://doi.org/10.17632/bm543vchc6.2 (https://data.mendeley.com/datasets/bm543vchc6/2) 3. K.B. Abdullahi, Statistical Mirroring Computer Application for Robust Dispersion Estimations, Mendeley Data V2 (2024). https://doi.org/10.17632/gzkkg2p68t.2 (https://data.mendeley.com/datasets/gzkkg2p68t/2)

## Method details

The method details are organized into four main sections as follows:

1. Introduction.
2. The literature review encompasses various aspects, including descriptions of estimators, the conceptual framework of Kabirian-based isomorphic optinalysis (including terminologies and definitions, theorems, and Python implementation), challenges and limitations associated with dispersion estimators, and the next-generation estimator.
3. The methodology of statistical mirroring is delineated through its definition, computational steps and algorithmic procedure, types of statistical mirroring, scale and scaloc-invariant statistical mirroring, general properties, Python implementation, drawbacks and limitations, and manual calculation.
4. Method application, validation, and comparison involve methods comparison using artificial datasets (including description of estimators, artificial datasets generation, data analysis and comparison, estimates and performance evaluation of the estimators), methods comparison under temperature measurement scales, results (including transformation invariance, efficiency, relative efficiency, sensitivity to location shift contaminations, sensitivity scaling contaminations, dispersion estimators under temperature measurement scales, set duplication invariance), discussion (including similar aspects as in results), conclusion, and recommendations.

## Introduction

Statistical analysis relies heavily on dispersion estimation, a fundamental pillar that illuminates the variability inherent within datasets, transcending mere measures of central tendency and underpinning decision-making processes across diverse fields and disciplines. From finance to healthcare, the significance of dispersion analysis resonates deeply, guiding risk assessments, performance evaluation, quality control, forecasting, benchmarking, and driving policy formulations [25], [26], [27], [28],30].

This study addresses some inherent challenges in existing dispersion estimation methods. Notably, classical efficient methods, categorized as mean deviation-based (such as variance, standard deviation, coefficient of variation, mean absolute deviation from mean, etc.) lack invariance to both location shift and scaling transformations, exhibit sensitivity to outliers, and limited applicability with skewed and asymmetric data types. Additionally, limitations include a trade-off between efficiency and robustness [1,^2^,^21^,[23], [24], [25], [26], [27], [28], [29], [30], [31].

Over the decades, attempts to provide robust efficient alternatives over the classical mean deviation-based estimators involved the establishment of methods such as median deviation-based (such as median absolute deviation from the median, Sn estimator by Rousseeuw-Croux, etc.), quartile deviation-based (such as interquartile range, quartile coefficient of dispersion, Qn estimator by Rousseeuw-Croux, etc.), and decile deviation-based (such as interdecile range, etc.) methods [4], [5], [6],30], alongside data modification techniques like winsorization and trimming [7], [8],30]. However, most of these robust approaches often sacrifice efficiency [4], [5], [6], [7], [8],30]. Despite attempts to mitigate these trade-offs through techniques like winsorization and trimming, a comprehensive solution remains elusive [[7], [8], [10]].

Recent methodological advances in the Kabirian-based optinalysis model mark a turning point in addressing these longstanding issues [9]. This model, known for its customization and adaptability [9], presents an opportunity to create alternative estimators for statistical dispersion. This study seeks to capitalize on this customization potential by proposing a novel methodology that addresses existing methodological limitations and offers a paradigm shift in estimation methodology.

The primary objective of this study is to propose statistical mirroring: a new and robust methodology for statistical dispersion estimation, offering a nuanced understanding of their behavior and properties. The methodology involves preprocessing transformations, statistical mirror design, and optimization to transform a univariate set into a bivariate one, facilitating the fitting of an isomorphic optinalysis model. These estimators rely on a foundational bijective mapping of isoreflective pairs, deducing the probability of proximity or deviation from any defined center—a departure from classical estimators that rely on average or median deviations from a mean or median center.

While the methodological breadth includes various types of statistical mirroring, the study specifically focuses on statistical meanic mirroring. Reference classical estimators, such as standard deviation and coefficient of variation, were chosen based on their efficiency and consideration of all data points around a mean center, representing a category of location-invariant and scale-invariant estimators [1,^2^,^9^,^12^,^30^,31]. The proposed and highlighted statistical meanic mirroring demonstrates heightened resistance to outliers and competing efficiency, presenting a significant contribution to statistical methodologies.

The significance of this study lies in its potential to advance dispersion estimation, offering a comprehensive and transformative framework that addresses longstanding challenges in statistical analysis. It endeavors to craft a sophisticated tool that navigates the complexities of modern data analysis, offering invaluable insights across a myriad of domains and applications. Moreover, it recommends further exploration of proposed estimators across various statistical mirroring types and encourages comparative studies to establish their effectiveness, thereby advancing statistical knowledge and enhancing analytical capabilities across diverse domains.

## Literature review

This overview delves into the world of statistical dispersion estimators, categorizing them into distinct methods and highlighting their unique characteristics. From mean and median deviation-based approaches to quartile and decile deviation-based methods, the discussion explores their framework, efficiency, robustness, sensitivity to outliers, and transformative invariances. The discourse also outlines challenges associated with existing estimators. Looking forward, it suggests key properties for the next-generation estimator, addressing current limitations and fostering adaptability to diverse data types. This concise guide serves as a valuable resource for researchers and practitioners navigating the complexities of dispersion analysis, urging a thoughtful selection based on data nuances and research objectives.

### Description of estimators

Table 1 describes some common estimators of statistical dispersions, highlighting some of their key characterized properties and limitations.

**Table 1:** Properties and limitations of some common estimators of statistical dispersions.

Estimator	Statistical framework	Efficiency	Outliers	Probabilistic range	Transformation invariance	*Scaloc-Invariance*	References
Variance	Classical parametric	Highly efficient	Sensitive	No	Location shift-invariant	No	[1]
Standard Deviation	Classical parametric	Highly efficient	Sensitive	No	Location shift-invariant	No	[1]
Coefficient of Variation (*CV*)	Classical parametric	Highly efficient	Sensitive	No	Scale-invariant	No	[2]
Mean Absolute Deviation from Mean (MAD)	Robust nonparametric	Less efficient	Resistant	No	Location shift-invariant	No	[1,3]
Median Absolute Deviation from the Median	Robust nonparametric	Less efficient	Resistant	No	Location shift-invariant	No	[1,3]
Sn Estimator	Robust nonparametric	Efficient	Resistant	No	Location shift-invariant	No	[4]
Interquartile Range (IQR)	Robust nonparametric	Less efficient	Resistant	No	Location shift-invariant	No	[1,5]
Quartile Coefficient of Dispersion	Robust nonparametric	Not discussed	Resistant	No	Scale-invariant	No	[5]
Qn Estimator	Robust nonparametric	Efficient	Resistant	No	Location shift-invariant	No	[4]
Interdecile Range	Robust nonparametric	Less efficient	Resistant	No	Location shift-invariant	No	[6]
Trimmed Mean Deviation (TMD)	Robust nonparametric	Less efficient	Resistant	No	Location shift-invariant	No	[7]
Winsorized Variance	Robust nonparametric	Less efficient	Resistant	No	Location shift-invariant	No	[8]
Winsorized Standard Deviation	Robust nonparametric	Less efficient	Resistant	No	Location shift-invariant	No	[8]

*Note:* The word “*scaloc*” from “*scaloc-invariance*” is a coined hybridization of two words: scale and location transformations.

The following categories highlight the diverse estimators in statistical dispersion estimations, offering practitioners a range of options based on the characteristics of their data and the goals of the analysis [1], [2], [3], [4], [5], [6], [7], [8],30].

1. Mean deviation-based methods: The mean deviation-based estimators focus on measures derived from deviations of individual data points from a central tendency, often the mean. Variance and Standard Deviation are classical parametric estimators that quantify the dispersion by squaring deviations and taking their square root, respectively. The Coefficient of Variation (*CV*) adds a scaling factor, providing a dimensionless measure. Mean Absolute Deviation from Mean (MAD) directly uses the absolute deviations. These estimators are very efficient but sensitive to outliers [1], [2], [3],30].
2. Median deviation-based methods: Median deviation-based estimators, such as Median Absolute Deviation from the Median and the Sn Estimator by Rousseeuw-Croux, derive measures of dispersion using the median as a robust central location estimate. These estimators are less efficient but highly resistant to outliers, making them suitable for skewed datasets. The Sn by Rousseeuw-Croux has been a modified more efficient median deviation-based estimator [1,^3^,^4^,30].
3. Quartile deviation-based methods: Quartile deviation-based estimators, including the Interquartile Range (IQR), Quartile Coefficient of Dispersion, and Qn Estimator by Rousseeuw-Croux, focus on the quartiles of the data distribution. IQR measures the range between the first and third quartiles, while the Quartile Coefficient of Dispersion and Qn Estimator introduce robustness. These estimators are resistant to outliers and less efficient. The Qn by Rousseeuw-Croux has been a modified more efficient median deviation-based estimator [1,^4^,^5^,30].
4. Decile deviation-based methods: Decile deviation-based methods, represented by the Interdecile Range, consider the spread between deciles of the dataset. Like other robust estimators, the Interdecile Range is resistant to outliers and less efficient [6,30].
5. Data modification techniques: Data modification techniques, such as Winsorization and Trimming, involve adjusting extreme values in the dataset. Winsorized Variance and Winsorized Standard Deviation are examples of estimators that result from Winsorization. These methods aim to enhance robustness and reduce the influence of outliers, providing alternative estimates of dispersion. These estimators are very less efficient [7], [8],30].

### Conceptual framework of Kabirian-based isomorphic optinalysis

Defining the conceptual framework of Kabirian-based isomorphic optinalysis is associated with some unique definitions of terms. These terms and definitions were introduced for the first time or conceptually modified and form the basis of the methodological novelties of the Kabirian-based isomorphic optinalysis [9]. The following are some of the relevant terms used. They were presented in this paper as defined or explained by the original author.

Definition 1 Theoretical ordering

“Theoretical ordering refers to theory-based, or concept-based structuring or arrangement of terms and items. For instance, the arrangement of real numbers in ascending or descending order is theory-based” [9].

Definition 2 Isoreflective pair

“An isoreflective pair describes a concatenated mirror isomorphism between two mathematical structures about a center. Let $A=(a_{1},a_{2},\;a_{3},\ldots ,a_{n})$ and $B=(b_{1},b_{2},\;b_{3},\ldots ,b_{n})$ two mathematical structures” [9]. “Then, the isoreflective pair is represented as:



Such that δ∉A,B; δ,A,B∈R.”

Remark 2 “The standard notation to represent mirror isomorphism, A≅B or A→B, is modified as  *B* to emphasize a center δ as an important term, as well as the concatenation of the pair [9].”

Definition 3 Head-to-head reflection or pairing

“A reflection or pairing of the isoreflective pair is head-to-head if the first terms (elements) of the isoreflective pair are maximally distant from the central connection point [9]. Let $A=(a_{1},a_{2},\;a_{3},\ldots ,a_{n})$ and $B=(b_{1},b_{2},\;b_{3},\ldots ,b_{n})$ be two mathematical structures about a center δ. Then, head-to-head isoreflective pairing is represented as:



Such that δ∉A,B; δ,A,B∈R [9].”

Definition 4 Tail-to-tail reflection or pairing

“A reflection or pairing of an isoreflective pair is head-to-head if the first terms (elements) of the isoreflective pair are minimally distant (i.e., positioned at their closest proximity) from the central connection point [9].” “Let $A=(a_{1},a_{2},\;a_{3},\ldots ,a_{n})$ and $B=(b_{1},b_{2},\;b_{3},\ldots ,b_{n})$ two mathematical structures about a center δ. Then, tail-to-tail isoreflective pairing is represented as:



Such that δ∉A,B; δ,A,B∈R [9].”

Definition 5 Pericentral rotation

“Pericentral rotation refers to the turning of all the members of two mathematical structures of an isoreflective pair through 180° around the pericentres [9].

A pericentre is the median point of each mathematical structure. Pericentral rotation is similar to alternate reflection (i.e., from the head-to-head to tail-to-tail reflection or otherwise). An alternate reflection is the alternative form of reflection between isoreflective pairs” [9].

Definition 6 Central rotation

“Central rotation refers to the turning of all the members of two mathematical structures of an isoreflective pair through 180° around the central point. Central rotation is similar to inversion transformation" [9].

“Let  be a tail-to-tail isoreflective pair of two mathematical structures around a central (δ), such that δ∉A,B; δ,A,B∈R

Then, its central rotation or inversion becomes  [9]”

Definition 7 Optiscale

“Optiscale (denoted as R) is a subset of either the positive real numbers (excluding zero) or the negative real numbers (excluding zero). The optiscale consists of numbers that can be represented as multiples of a positive constant k, where k represents the uniform interval between the numbers in the scale” [9]. “The notation used to represent the optiscale is as follows:

For the subset of positive real numbers:

$$R\subseteq \left\{r\in R^{+*}|r=n*k,\;n\in N,\;k>0\right\}$$

For the subset of negative real numbers:

$$R\subseteq \left\{r\in R^{-*}|r=-n*k,\;n\in N,\;k>0\right\}$$

In both cases, $R^{+*}$ represents the set of positive real numbers (excluding zero),$R^{-*}$ represents the set of negative real numbers (excluding zero), N represents the set of natural numbers (positive integers), and n is a natural number that acts as a multiplier for k. The optiscale includes all numbers that can be obtained by multiplying the positive constant k by a natural number n [9].”

Definition 8 Optinalysis

“Optinalysis is a function that autoreflectively or isoreflectively compares the symmetry/asymmetry, similarity/dissimilarity, and identity/unidentity within one or between two mathematical structures as a mirror-like (optic-like) reflection of each other about a central point. In other words, it is a function that numerically compares isoreflective or autoreflective pairs of mathematical structures” [9].

**Fig. 1:**
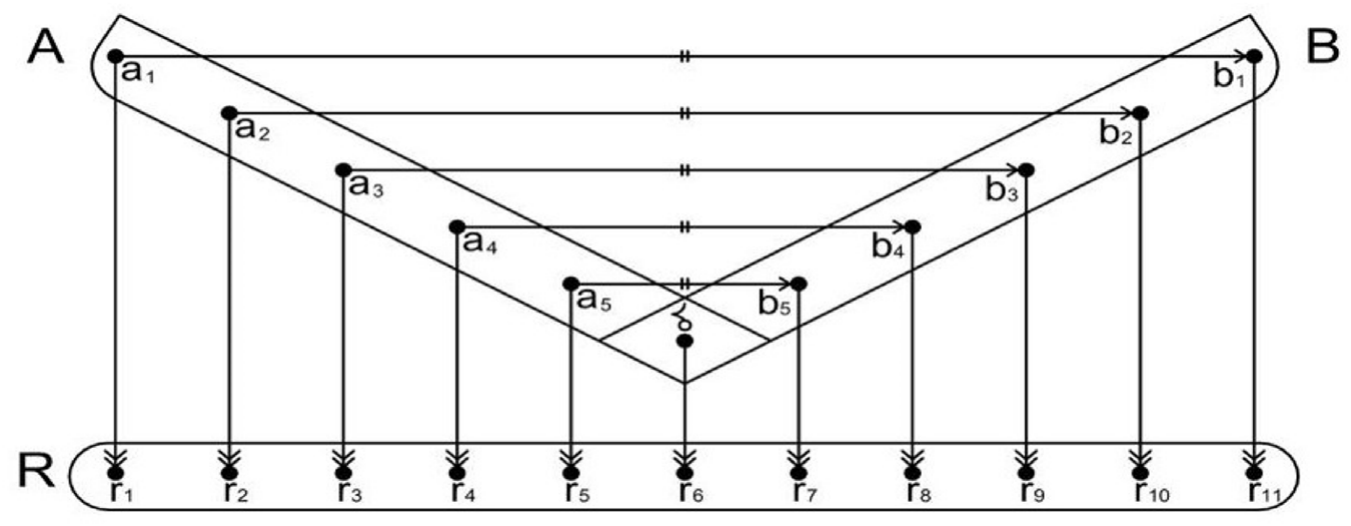
“Linear mapping between an isoreflective pair of points and linear *re-mapping* with the optiscale. A represents the domain, while B represents the co-domain of A. δ denotes a central point, and R represents the optiscale. The symbol  indicates a bijective mapping between the isoreflective pair around a central point, and  indicates a linear re-mapping with the optiscale R” [9].

“Optinalysis is a function that is comprised of an assigned optiscale (R) that bijectively re-maps (a symbol  indicates a re-mapping) an isoreflective pair of mathematical structures. Fig. 1 illustrates how isoreflective pairs of points are mapped and also re-mapped with an optiscale. Optinalysis is expressed in *optinalytic construction*” [9].

Definition 9 Isomorphic optinalysis:

“Isomorphic or comparative optinalysis refers to the analysis of isoreflective pairs of mathematical structures by optinalysis. It is a method of similarity/dissimilarity and identity estimation. Comparative optinalysis is defined by its optinalytic construction as follows” [9]:

“Let P be an isoreflective pair of mathematical structures A and B about a center δ. Let  indicate a linear re-mapping. Let R be the assigned optiscale. Then, isomorphic optinalysis as a function is defined as” [9]:

f:P→R

“The isoreflective pair P of the two mathematical structures A B has been defined as . Now optiscale R is introduced into the function to establish a linear re-mapping with the P. We now have new representations (called optinalytic constructions):



Such that δ∉A,B;A,B,δ∈R;$R\subseteq \{r\in R^{+*}|r=n*k,\;n\in N,\;k>0\}$ or alternatively $R\subseteq \{r\in R^{-*}|r=-n*k,\;n\in N,\;k>0\}$; n∈N; and A&B are isoreflective pairs on a chosen pairing about a central point δ [9].”

Definition 10 Scalement

“A *scalement* refers to the product of any member of an autoreflective or isoreflective pair of mathematical structures and its assigned optiscale” [9].

“Let the optinalytic construction of an isoreflective pair of two mathematical structures A and B with an assigned optiscale (R) be:



Such that δ∉A,B;A,B,δ∈R;$R\subseteq \{r\in R^{+*}|r=n*k,\;n\in N,\;k>0\}$ or alternatively $R\subseteq \{r\in R^{-*}|r=-n*k,\;n\in N,\;k>0\}$; n∈N; and A&B are isoreflective pairs on a chosen pairing about a central point δ.”

“Then, the sum of scalements S of the isoreflective pair between mathematical structures A and B is defined as” [9]:

$$S\left(A,B\right)=\left(r_{1}.a_{1}\right)+\ldots +\left(r_{n+1}.\delta \right)+\ldots +\left(r_{2n+1}.b_{1}\right)=\sum_{i=1}^{n}\sum_{j=k=n+2}^{2n+1}\left(r_{i}a_{i}+r_{n+1}\delta +r_{j}b_{i}\right)$$

Definition 11 Kabirian coefficient of isomorphic optinalysis

“The level of similarity or identity of an isoreflective pair of mathematical structures under optinalysis is defined by the optinalytic coefficient, known as the Kabirian coefficient (Kc). The Kabirian coefficient for isomorphic optinalysis is expressed as the quotient of the product of the median optiscale and the summation of all elements (of the isoreflective pair) divided by the summation of all scalements (of the isoreflective pair). Kabirian coefficient of isomorphic optinalysis is proven to be functionally operating based on isomorphism (i.e., a bijective mapping between an isoreflective pair of two mathematical structures). Find the detail of the mathematical proof in Abdullahi” [9].

“Let the optinalytic constructions of isoreflective pair of two mathematical structures A and B with an assigned optiscale (R) be:

Such that δ∉A,B;A,B,δ∈R;$R\subseteq \{r\in R^{+*}|r=n*k,\;n\in N,\;k>0\}$ or alternatively $R\subseteq \{r\in R^{-*}|r=-n*k,\;n\in N,\;k>0\}$; n∈N; and A&B are isoreflective pairs on a chosen pairing about a central point δ [9].”

“Then, the Kabirian coefficient of similarity or identity between the isoreflective pair is expressed by Eq. (1) [9]”.

(1) $$KC_{Sim.\;/Id.}\left(A,B\right)=\frac{r_{n+1}\left(a_{1}+a_{2}+a_{3}+\ldots +a_{n}+\;\delta +b_{n}+\ldots +b_{3}+b_{2}+b_{1}\right)}{\begin{array}{c} \left(r_{1}.a_{1}\right)+\left(r_{2}.a_{2}\right)+\left(r_{3}.a_{3}\right)+\ldots +\left(r_{n}.a_{n}\right)+\left(r_{n+1}.\delta \right)+\\ \left(r_{n+2}.b_{n}\right)+\ldots +\left(r_{2n-1}.b_{3}\right)+\left(r_{2n}.b_{2}\right)+\left(r_{2n+1}.b_{1}\right) \end{array}}$$

(1a) $$KC_{Sim.\;/Id.}\left(A,B\right)=\frac{r_{n+1}\left[\sum_{i=1}^{n}\left(a_{i}+\delta +b_{i}\right)\right]}{\sum_{i=1}^{n}\sum_{j=n+2}^{2n+1}\left(r_{i}a_{i}+r_{n+1}\delta +r_{j}b_{i}\right)}$$

$$\left\{ \begin{array}{c} if\;g\left(A\right)=g\left(B\right);\;then\;KC_{Sim.\;/Id.}\left(A,B\right)=1\\ if\;g\left(A\right)=-g\left(B\right),\;or\;-\;g\left(A\right)=g\left(B\right);\;then\;KC_{Sim.\;/Id.}\left(A,B\right)=0\\ if\;g\left(A\right)<g\left(B\right);\;then\;0\leq \;KC_{Sim.\;/Id.}\left(A,B\right)\leq 1\\ if\;g\left(A\right)>g\left(B\right);\;then\;1\leq KC_{Sim.\;/Id.}\left(A,B\right)\leq n+1\\ if\;g\left(A\right)>g\left(B\right);\;then\;KC_{Sim.\;/Id.}\left(A,B\right)\geq n+1,<0 \end{array}\right.$$

Definition 12 Kabirian-based optinalysis-to-probability translation models

“The Kabirian-based optinalysis-to-probability translation models are bridges that connect the outcomes of Kabirian-based optinalysis (i.e., Kabirian bi-coefficients) to probability. The translation models translate the two possible Kabirian bi-coefficients into a probability that infers the level of certainty to which the isoreflective pair of mathematical structures are similar, identical, or otherwise” [9].

#### Phase 1: forward translation, from Kabirian bi-coefficients to probability of similarity [9]

“Eq. (2) translates forward the Kabirian coefficient of similarity and identity ($KC_{Sim./Id.}$) between isoreflective pair of mathematical structures under Kabirian-based optinalysis to the probability of similarity and identity ($P_{Sim./Id.}$) [9].”

(2) $$P_{Sim./Id.}\left(A,B\right)=\frac{\left(nr_{1}+r_{1}\right)-K_{c}\left(2nr_{1}+r_{1}\right)}{r_{1}\times K_{c}-\left(nr_{1}+r_{1}\right)},\;\forall \;0\leq K_{c}\leq 1$$

$$\left\{ \begin{array}{c} if\;\frac{n+1}{2n+1}\leq \;KC_{Sim./Id}\left(A,B\right)\leq 1;\;then\;0\leq P_{Sim./Id.}\left(A,B\right)\leq 1\\ if\;0\leq \;KC_{Sim./Id.}\left(A,B\right)\leq \frac{n+1}{2n+1};\;then\;-1\leq P_{Sim./Id.}\left(A,B\right)\leq 0 \end{array}\right.$$

Or inversely as:

(3) $$P_{Sim./Id.}\left(A,B\right)=\frac{\left(nr_{1}+r_{1}\right)-r_{1}K_{c}}{\left(2nr_{1}+r_{1}\right)K_{c}-\left(nr_{1}+r_{1}\right)},\;\forall \;1\leq K_{c}\leq n+1;K_{c}\geq n+1;\;\&K_{c}\leq 0$$

$$\left\{ \begin{array}{c} if\;1\leq \;KC_{Sim./Id.}\left(A,B\right)\leq n+1;\;then\;0\leq P_{Sim./Id.}\left(A,B\right)\leq 1\\ if\;KC_{Sim./Id.}\left(A,B\right)\geq n+1,\;or\;\leq 0;\;then\;-1\leq P_{Sim./Id.}\left(A,B\right)\leq 0 \end{array}\right.$$

#### Phase 2: forward translation, from the probability of similarity, and identity to the probability of dissimilarity, and unidentity [9]

“(3), (4) translate forward the probability of similarity and identity ($P_{Sim./Id.}$) to the probability of dissimilarity, and unidentity ($P_{Dsim./Uid.}$) between isoreflective pair of mathematical structures under Kabirian-based optinalysis. Translation of the Kabirian coefficient is valid if and only if the outcomes are within the range of values −1 to 1 (or −100 to 100 of its equivalent percentage). [9]”

If $P_{Sim./Id.}\left(A,B\right)\geq 0$, then

(4) $$P_{Dsim.\;/Uid.}\left(A,B\right)=1-P_{Sim./Id.}\left(A,B\right)$$

If $P_{Sim./Id.}\left(A,B\right)\leq 0$, then

(5) $$P_{Dsim.\;/Uid.}\left(A,B\right)=-1-P_{Sim./Id.}\left(A,B\right)$$

#### Phase 3: backward translation: from the probability of dissimilarity, and unidentity to the probability of similarity, and identity [9]

“(5), (6) translate backward the probability of dissimilarity and unidentity ($P_{Dsim./Uid.}$) to the probability of similarity, and identity ($P_{Sim./Id.}$) respectively [9].”

If $P_{Dsim./Uid.}\left(A,B\right)\geq 0$, then

(6) $$P_{Sim.\;/Id.}\left(A,B\right)=1-P_{Dsim./Uid}\left(A,B\right)$$

If $P_{Dsim./Uid.}\left(A,B\right)\geq 0$, then

(7) $$P_{Sim.\;/Id.}\left(A,B\right)=-1-P_{Dsim./Uid.}\left(A,B\right)$$

#### Phase 4: backward translation: from the probability of similarity, and identity to Kabirian bi-coefficients [9]

“These (7), (8) translate backward the probability of similarity, and identity outcomes to its two possible Kabirian bi-coefficients, designated as KC_Alt.1 and KC_Alt.2.

(8) $$KC\_Alt.1\left(A,B\right)=\frac{\left(nr_{1}+r_{1}\right)\left(P_{Sim./Id.}+1\right)}{\left(r_{1}\times P_{Sim./Id}\right)+\left(2nr_{1}+r_{1}\right)},\;\forall \;0\leq K_{c}\leq 1$$

(9) $$KC\_Alt.2\left(A,B\right)=\frac{\left(nr_{1}+r_{1}\right)\left(1+P_{Sim./Id.}\right)}{r_{1}+P_{Sim./Id.}\left(2nr_{1}+r_{1}\right)},\;\forall \;1\leq K_{c}\leq n+1;K_{c}\geq n+1\;\&\;\forall \;K_{c}\leq 0$$

where $r_{1}$ is the first term of the established optiscale and n is the sample size/item length [9].”

“The expectations of this translation model (of forward and backward translations of Kabirian-based optinalytic outcomes) are described as *Y-rule* (of Kabirian-based isomorphic). The Y-rule demonstrated below, is a Y-shaped chain of forward and backward proceedings of Kabirian-based isomorphic outcomes” [9].



### Theorem and properties in Kabirian-based optinalysis

The theorems within Kabirian-based optinalysis exemplify its properties and are highlighted as follows:

i. Kabirian-based isomorphic optinalysis establishes a bijective connection between corresponding elements of two mathematical structures when represented as functions [9]. The complete statement of the theorem and its proof are detailed in Abdullahi [9].
ii. Completeness invariance in optinalysis ensures that estimates remain the same under various transformations such as rotation, reflection, translation, and modulation in completely similar structures [9]. The complete statement of the theorem and its proof are detailed in Abdullahi [9].
iii. Incompleteness invariance in optinalysis ensures that estimates remain unaffected by transformations like product translation and central rotation in incompletely similar structures [9]. The complete statement of the theorem and its proof are provided in Abdullahi [9].
iv. Optinalytic normalization mitigates the impact of incompleteness by employing central modulation, thereby achieving near-completeness in incompletely similar structures [9]. The complete statement of the theorem and its proof are available in Abdullahi [9].
v. Kabirian-based optinalysis-to-probability translation models estimate unknown probabilities of similarity using optinalysis coefficients and a predefined optiscale, establishing a relationship between actual and expected probabilities [9]. The complete statement of the theorem and its proof are elaborated in Abdullahi [9].

### Python implementation

The Python code for Kabirian-based isomorphic optinalysis is available at Abdullahi [13] or via these links: https://data.mendeley.com/datasets/gnrcj8s7fp/2.

### The challenges and limitations associated with dispersion estimators

Understanding the challenges associated with dispersion estimators is crucial for researchers and practitioners when selecting dispersion estimators for their analyses. It emphasizes the need for a thoughtful and context-specific choice based on the unique characteristics of the data and the goals of the study.

The challenges and limitations associated with dispersion estimators can be further elaborated upon as follows:

a. A trade-off between robustness and efficiency: Classical estimators, such as Variance and Standard Deviation, and mean deviation-based estimators often present a trade-off between robustness and efficiency. Classical estimators may be highly efficient but are sensitive to outliers, sacrificing robustness. On the other hand, robust estimators, like Median Absolute Deviation from the Median, Interquartile Range, Quartile Coefficient of Dispersion, interdecile range, or the Qn and Sn Estimators, and those estimators that employ trimming and winsorization modifications of data, prioritize resistance to outliers over efficiency. The choice of which estimator to use depends on the specific characteristics of the data and the research objectives [1], [2], [3],^23^,^24^,26].
b. Interpretation challenges without probabilistic bounded range: A common limitation across all estimators is the lack of a probabilistic bounded range. The absence of such a range complicates the interpretation of results. Users are unable to quantify the uncertainty associated with the estimates, making it challenging to communicate the reliability of the findings. A probabilistic range provides a more nuanced understanding of the variability inherent in the estimates, aiding in decision-making and risk assessment [3,^19^,30].
c. *Scaloc-invariance* properties: Another notable limitation is the absence of scaloc-invariance properties in the estimators. This means that the estimators cannot be compared across datasets with combined location shifts and scaling transformations. For instance, comparing temperature scales which are based on different intervals or ratios becomes problematic. The inability to achieve scaloc invariance restricts the generalizability and applicability of the estimators, particularly in contexts where datasets are transformed and measured on different scales [31].
d. Limited applicability to diverse data types: Dispersion estimators, while versatile, may not perform optimally across all types of data. Certain estimators may be better suited for symmetric datasets, while others excel in handling skewed or heavy-tailed distributions. Users must carefully consider their data's distributional properties to select an appropriate estimator. This limitation emphasizes the importance of understanding the underlying data characteristics before applying dispersion estimators [4,^5^,25].
e. Impact of extreme values on certain estimators: Some dispersion estimators, especially those based on mean deviations or squared mean deviations, can be heavily influenced by extreme values. Outliers can disproportionately affect the estimates, potentially leading to biased results. Robust estimators aim to mitigate this impact, but the choice of a specific robust estimator should align with the degree of robustness required for the analysis [4,^23^,^25^,30].
f. Assumption of symmetry: Several traditional dispersion estimators assume symmetry in the underlying distribution. This assumption may not hold in real-world scenarios where data distributions exhibit skewness or asymmetry. In such cases, the estimators may not accurately capture the dispersion characteristics of the data, affecting the validity of the results [25,^26^,28].
g. Computational complexity: Some robust estimators, especially those based on complex algorithms or iterative procedures, may exhibit higher computational complexity compared to simpler classical estimators. This can pose challenges in terms of computational resources and processing time, particularly when dealing with large datasets [16,^23^,25].

### Next-generation estimator

Designing the next-generation estimator involves addressing the challenges posed by existing estimators while incorporating properties that enhance versatility, interpretability, and robustness. Here are some key properties that the next proposed estimator could possess to be considered an advancement over existing ones:

i. Robust efficiency: Achieving a balance between robustness and efficiency is crucial. The next estimator should demonstrate robustness against outliers while maintaining efficiency in estimating dispersion. This could involve developing adaptive methods that dynamically adjust to the data characteristics, providing accurate estimates without compromising robustness [8,^12^,^12^,^22^,30].
ii. Probabilistic bounded range: An improved estimator should introduce a probabilistic bounded range, offering a measure of uncertainty around the estimated dispersion. This feature enhances the interpretability of results and provides decision-makers with a more comprehensive understanding of the reliability of the estimates [19,30].
iii. Scaloc-invariance properties: Overcoming the limitation of lacking *scaloc-invariance*, the next estimator should ideally possess properties that allow for the comparison of datasets with different location shifts and scaling transformations. This is particularly important in fields where measurements are conducted on different scales or involve diverse units of measurement.
iv. Adaptability to diverse data types: The proposed estimator should be versatile enough to handle a wide range of data types, including symmetric, skewed, or heavy-tailed distributions. An adaptive approach that tailors the estimation method to the specific characteristics of the data could improve the estimator's applicability across diverse datasets [26], [27], [28], [29].
v. Resistance to the impact of extreme values: Minimizing the impact of extreme values on the estimator is essential. The next-generation estimator should employ techniques to robustly handle outliers without unduly influencing the results. This could involve advanced statistical methods or data modification strategies that appropriately down-weight the influence of extreme values [23,^25^,30].
vi. Non-symmetry assumption: Acknowledging the asymmetry often present in real-world data, the new estimator should not rely heavily on assumptions of symmetry. Designing methods that work well with both symmetric and asymmetric distributions ensures broader applicability and accuracy in diverse contexts [5,^25^,30].
vii. Computational efficiency: Considering the computational complexity associated with certain robust estimators, the next estimator should strike a balance between computational efficiency and accuracy. This involves developing efficient, scalable algorithms for large datasets, that and applicable in real-time or resource-constrained environments [16,28].
viii. Generalizability and consistency: The proposed estimator should be designed to generalize well across various statistical settings and maintain consistency in its performance. This ensures that the estimator's properties remain robust and reliable across different datasets and applications [25,31].
ix. Interpretability and user-friendliness: Enhancing the interpretability of the estimator's results is essential. Providing clear and intuitive outputs, along with appropriate measures of uncertainty, contributes to user-friendly interpretation. This can facilitate broader adoption across a range of users, including those without advanced statistical expertise [19,28].

Developing an estimator with these properties requires a combination of advanced statistical methods, computational techniques, and a deep understanding of the challenges faced by existing estimators. Collaboration between statisticians, data scientists, and domain experts is crucial for developing and implementing such a future estimator.

In summary, the exploration of dispersion estimators highlights the intricate considerations essential for robust statistical analysis. While current methods offer valuable insights, persistent challenges, such as the robustness-efficiency trade-off and interpretational limitations, call for innovative solutions. The envisioning of a future estimator emphasizes a crucial addition—the establishment of a scaloc-invariant category. This evolution seeks to reconcile the limitations in comparing datasets with different transformations, ensuring broader applicability across diverse scales. Collaborative efforts across disciplines are key to crafting a sophisticated tool that navigates complexities, striking a harmonious balance between robustness, adaptability, and interpretability in the ever-evolving landscape of statistical dispersion analysis.

## Methodology of statistical mirroring

The methodology applied in this study draws inspiration from the Kabirian-based isomorphic optinalysis proposed by Abdullahi [9]. Statistical mirroring encompasses preprocessing transformations, the design of a statistical mirror, and optimization aimed at transmuting a univariate set into a bivariate one, facilitating the fitting of an isomorphic (isoreflective) optinalysis model. The proposed estimator's statistical goodness was validated by comparing its performance with standard deviation and coefficient of variation.

In this section, several distinctive terms, including *principal value, statistical mirror, meanic, medianic, modalic, maximalic, minimalic, scaloc, endo-statistical*, and *exo-statistical* have been introduced. These terms are not widely recognized in existing literature but are purposefully coined or conceptually modified to underpin the methodological innovations presented in this research. They serve as key elements in conveying unique concepts and contribute to the foundation of novel methodologies developed in this study.

### Definition

Statistical mirroring is the measure of the proximity or deviation of transformed data points from a specified location estimate within a given distribution. Within the framework of Kabirian-based isomorphic optinalysis, statistical mirroring is conceptualized as the isoreflectivity (i.e., isoreflective pairing) of the transformed data points to a defined statistical mirror. This statistical mirror is an amplified location estimate of the transformed distribution, achieved through a specified size or length. The location estimate may include parameters such as the mean, median, mode, maximum, minimum, or reference value.

The process of statistical mirroring comprises two distinct phases:

(a) *Preprocessing phase:* This involves applying preprocessing transformations, such as compulsory theoretical ordering, with or without centering the data. It also encompasses tasks like statistical mirror design and optimizations within the established optinalytic construction. These optimizations include selecting an efficient pairing style, central normalization, and establishing an isoreflective pair between the preprocessed data and its designed statistical mirror.
(b) *Optinalytic model calculation phase:* This phase is focused on computing estimates (such as the Kabirian coefficient of proximity, the probability of proximity, and the deviation) based on Kabirian-based isomorphic optinalysis models.

### Computational steps and algorithmic procedure

Let $X=(x_{3},x_{1},x_{2},.\ldots .,x_{n})$ be a random variable. Statistical mirroring involves the following steps

*Preprocessing phase*

Let the order of algorithmic transformations $t_{c}$ and $t_{o}$ as centering and ordering of the variable X respectively.

*Step 1:* Centering the variable X (i.e., location removal), which is optional based on the task at hand. Centering of the variable can be by mean, median, mode, maximum, minimum, reference value, or other operations.
  $$t_{c}\left(X\right)=\sum_{i=1}^{n}\left(X_{i}-\dddot{X}\right)$$

Where $\dddot{X}$ is the mean, median, mode, maximum, minimum, reference value, etc. of the variable X. For efficiency and the specific task at hand, the absolute positive (transforming to purely positive values) or absolute negative (transforming to purely negative values) distances can be returned.

$$t_{c}\left(X\right)=\left(x_{3},x_{1},x_{2},.\ldots .,x_{n}\right)$$

*Step 2:* Establish a compulsory theoretical order for the $t_{c}\left(X\right)$ variable. Note that numerical values are theoretically arranged in ascending or descending order. This ordering ensures permutation-invariance of the estimators.
  $$t_{c\to o}\left(X\right)=\left(x_{1}\leq ,x_{2},\leq \;x_{3},\leq .\ldots .,\leq x_{n}\right)$$

or alternatively

$$t_{c\to o}\left(X\right)=\left(x_{1}\geq ,x_{2},\geq x_{3},\geq .\ldots .,\geq x_{n}\right)$$

*Step 3:* Design an efficient statistical mirror. A statistical mirror refers to a defined and amplified location estimate, called principal value (e.g., mean, median, mode, maximum, minimum, reference value, etc.) of the variable through a defined length. Therefore, different types of statistical mirrors can be designed, but the choice depends on the task to be performed.
  $$P=\left[p\right]*n=\left(p_{1},p_{2},p_{3},.\ldots .,p_{n}\right)$$

Such that the principal value is the $p=g(t_{c\to o}\left(X\right))$, p∈P, P is the statistical mirror, n∈N, X∈R, and the g function is the defined location estimates.

Step 4: Establish and optimize the optinalytic construction. Note that optinalytic construction is the final step before isomorphic optinalysis. In this step, choosing an efficient pairing style (reflection) and establishing an isoreflective pair between $t_{c\to o}\left(X\right)$ onto P about δ are the main focus. For instance:

Head-to-head pairing or reflection of the isoreflective pair is given as:



Or in a choice, the tail-to-tail pairing or reflection of the isoreflective pair is given as:



Such that $t_{c\to o}\left(X\right),P,\;\delta \;\&\;R\in R$; $r_{1}\neq 0$; n∈N; R is the optiscale; and $t_{c\to o}\left(X\right)\;\&\;P$ are isoreflective pairs. δ=0 is by default operation, except under optinalytic normalization.

#### Optinalytic model calculation phase

Step 5: Using the Kabirian-based isomorphic optinalysis models [9,13], estimate the Kabirian coefficient of statistical proximity/similarity $\left(KC_{Sprox.}\right)$, probability of statistical proximity/similarity $\left(P_{Sprox.}\right)$, and other derivative estimates, which satisfied the Y-rule of Kabirian-based isomorphic optinalysis.

 .

Where X,P∈R.

The two possible Kabirian bi-coefficients $\left(KC1_{Sprox.}\;\&\;KC2_{Sprox.}\right)$ function on two different, but inverse optinalytic operations.

### Types of statistical mirroring

Let $t_{c\to o}\left(X\right)$ be a transformed random variable. Let optional centering and compulsory ordering transformations of the X variable be $t_{c}$ and $t_{o}$ respectively. Let $t_{c^{\mp }}$ and $t_{c^{+}}$ differentiate between a centering that does not return absolute values and a centering that returns absolute values respectively. Let the statistical mirror be P=[p]*n, and the principal value $p=g(t_{c\to o}\left(X\right))$. n is the sample size of the $t_{c\to o}\left(X\right)$ variable.

Suppose that  *R* defines a statistical mirroring, where R is the optinalytic optiscale. It is called:

a. *A statistical meanic mirroring*, if $p=M_{n}$. Where $M_{n}$ is the mean (the function) of the transformed variable $t_{c\to o}\left(X\right)$. It is a measure of proximity or deviation (how close or far) the transformed data points are from their mean estimate. It is further referring to a raw meanic mirroring if $M_{n}=g\left(t_{o}\left(X\right)\right)$, integral meanic mirroring if $M_{n}=g\left(t_{c^{\mp }\to o}\left(X\right)\right)$, and also an absolute meanic mirroring if $M_{n}=g\left(t_{c^{+}\to o}\left(X\right)\right)$.
b. *A statistical medianic mirroring*, if $p=M_{d}$. Where $M_{d}$ is the median value of the transformed variable $t_{c\to o}\left(X\right)$. It is a measure of proximity or deviation (how close or far) the transformed data points are from their median estimate. It is further referring to a raw medianic mirroring if $M_{d}=g\left(t_{o}\left(X\right)\right)$, integral medianic mirroring if $M_{d}=g\left(t_{c^{\mp }\to o}\left(X\right)\right)$, and also an absolute medianic mirroring if $M_{d}=g\left(t_{c^{+}\to o}\left(X\right)\right)$.
c. *A statistical modalic mirroring*, if $p=M_{o}$. Where $M_{o}$ is the modal value of the transformed variable $t_{c\to o}\left(X\right)$. It is the measure of proximity or deviation (how close or far) the transformed data points are from the modal estimate. It is further referring to a raw modalic mirroring if $M_{o}=g\left(t_{o}\left(X\right)\right)$, integral modalic mirroring if $M_{o}=g\left(t_{c^{\mp }\to o}\left(X\right)\right)$, and also an absolute modalic mirroring if $M_{o}=g\left(t_{c^{+}\to o}\left(X\right)\right)$.
d. *A statistical minimalic mirroring*, if $p=M_{x}$. Where $M_{x}$ is the minimum value of the transformed variable $t_{c\to o}\left(X\right)$. It is a measure of proximity or deviation (how close or far) the transformed data points are from the minimum estimate. It is further referring to a raw minimalic mirroring if $M_{x}=g\left(t_{o}\left(X\right)\right)$, integral minimalic mirroring if $M_{x}=g\left(t_{c^{\mp }\to o}\left(X\right)\right)$, and also an absolute minimalic mirroring if $M_{x}=g\left(t_{c^{+}\to o}\left(X\right)\right)$.
e. *A statistical maximalic mirroring*, if $p=M_{y}$. Where $M_{y}$ is the maximum value of the transformed variable $t_{c\to o}\left(X\right)$. It is a measure of proximity or deviation (how close or far) the transformed data points are from the maximum estimate. It is further referring to a raw maximalic mirroring if $M_{y}=g\left(t_{o}\left(X\right)\right)$, integral maximalic mirroring if $M_{y}=g\left(t_{c^{\mp }\to o}\left(X\right)\right)$, and also an absolute maximalic mirroring if $M_{y}=g\left(t_{c^{+}\to o}\left(X\right)\right)$.
f. *A statistical reference mirroring*, if $p=R_{f}$. Where $R_{f}$ is a reference value outside the transformed variable $t_{c\to o}\left(X\right)$. It is a measure of proximity or deviation (how close or far) the transformed data points are from the reference estimate value. It is further referring to a raw reference mirroring if $R_{f}=g\left(t_{o}\left(X\right)\right)$, integral reference mirroring if $R_{f}=g\left(t_{c^{\mp }\to o}\left(X\right)\right)$, and also an absolute reference mirroring if $R_{f}=g\left(t_{c^{+}\to o}\left(X\right)\right)$.
g. *An endo-statistical mirroring*, if $p=M_{n},\;M_{d},\;M_{o},\;M_{x},\;M_{y}$ of the location estimates of the transformed variable $t_{c\to o}\left(X\right)$.
h. *An exo-statistical mirroring*, if $p\neq M_{n},\;M_{d},\;M_{o},\;M_{x},\;M_{y}$ of the location estimates of the transformed variable $t_{c\to o}\left(X\right)$.

### Scale and scaloc-invariant statistical mirroring

Statistical mirroring is termed scale-invariant if the efficient location parameter is retained in the variable, while it is called scaloc-invariant if the efficient location parameter is removed. Note that “scaloc” is a hybridization of two words: scale and location.

### General properties of statistical mirroring

Statistical mirroring presents a versatile and robust approach for estimating dispersion, demonstrating its efficacy across various types of statistical mirrors and invariances. The proposed methodology exhibits applicability to both univariate and multidimensional datasets, making it a valuable tool for analyzing and interpreting diverse data distributions.

i. It is based on the entire observations of variables, unlike some robust statistics. Therefore, extreme maximum and minimum values are not discarded or trimmed.
ii. It applies to variable(s) from the set of real numbers.
iii. It has a unified and probabilistic bounded range for all dispersion estimates.
iv. It involves a measure of dispersion (i.e., proximity or deviation) from a defined location estimate (such as mean, median, maximum, minimum, and mode) and any other operation.
v. It is a permutation-invariant (i.e., remain unchanged even when the variable order is altered) estimator.
vi. It is a scale-invariant (i.e., robust to scale) estimator.
  Supposed we have an a scaling of a variable $X=(x_{1},x_{2},x_{3},.\ldots .,\;x_{n})$ and its statistical mirror $P=(p_{1},p_{2},p_{3},.\ldots .,p_{n})$, where P is the amplified estimate or reference value of the transformed variable X.
  $$KC_{Sprox.}\left(X,P\right)=KC_{Sprox.}\left(aX,P\right)$$
  $$P_{Sprox.}\left(X,P\right)=P_{Sprox.}\left(aX,P\right)$$
  $$P_{Sdev.}\left(X,P\right)=P_{Sdev.}\left(aX,P\right)$$
  where X,P,a∈R;a≠0.
vii. It is both scale-invariant and location-invariant estimator, provided that the efficient location parameter is either retained or removed, respectively, from the variable. For instance, taking the transformed distances from the mean.
  Supposed we have b location shift of a variable $X=(x_{1},x_{2},x_{3},.\ldots .,\;x_{n})$, and its statistical mirror $P=(p_{1},p_{2},p_{3},.\ldots .,p_{n})$, where P is the amplified estimate or reference value of the transformed variable X. It implies:
  $$KC_{Sprox.}\left(X,P\right)=KC_{Sprox.}\left\{\left(X+b\right)-\mu ,\;P\right\}\neq KC_{Sprox.}\left\{\left(X+b\right),\;P\right\}$$
  $$P_{Sprox.}\left(X,P\right)=P_{Sprox.}\left\{\left(X+b\right)-\mu ,\;P\right\}\neq P_{Sprox.}\left\{\left(X+b\right),\;P\right\}$$
  $$P_{Sdev.}\left(X,P\right)=P_{Sdev.}\left\{\left(X+b\right)-\mu ,\;P\right\}\neq P_{Sdev.}\left\{\left(X+b\right),\;P\right\}$$
  where X,P,b∈R; μ is the mean estimate of X+b.
viii. When both location and scale transformations are combined (e.g., Gaussian or linear transformation), it becomes scaloc-invariant, demonstrating robustness to both scale and location shifts.
  Supposed we have an a scaling and b location shift of a variable $X=(x_{1},x_{2},x_{3},.\ldots .,\;x_{n})$, and its statistical mirror $P=(p_{1},p_{2},p_{3},.\ldots .,p_{n})$, where P is the amplified estimate or reference value of the transformed variable X.
  $$KC_{Sprox.}\left(X,P\right)=KC_{Sprox.}\left\{\left(aX+b\right)-\mu ,\;P\right\}\neq KC_{Sprox.}\left\{\left(aX+b\right),\;P\right\}$$
  $$P_{Sprox.}\left(X,P\right)=P_{Sprox.}\left\{\left(aX+b\right)-\mu ,\;P\right\}\neq P_{Sprox.}\left\{\left(aX+b\right),\;P\right\}$$
  $$P_{Sdev.}\left(X,P\right)=P_{Sdev.}\left\{\left(aX+b\right)-\mu ,\;P\right\}\neq P_{Sdev.}\left\{\left(aX+b\right),\;P\right\}$$
  where X,P,a,b∈R; a≠0; μ is the mean estimate of Xa±b.
ix. It is variant to pericentral rotation (alternate reflection), except for statistical meanic mirroring.
  Supposed we have a variable $X=(x_{1},x_{2},x_{3},.\ldots .,\;x_{n})$ and its statistical mirror $P=(p_{1},p_{2},p_{3},.\ldots .,p_{n})$, where P is the amplified estimate or reference value of the transformed variable X.
  $$KC_{\mathrm{Sprox}.}\left(\overset{\leftarrow }{X}|\vec{P}\right)\neq KC_{\mathrm{Sprox}.}\left(\vec{X}|\overset{\leftarrow }{P}\right)$$
  $$P_{\mathrm{Sprox}.}\left(\overset{\leftarrow }{X}|\vec{P}\right)\neq P_{\mathrm{Sprox}.}\left(\vec{X}|\overset{\leftarrow }{P}\right)$$
  $$P_{\mathrm{Sdev}.}\left(\overset{\leftarrow }{X}|\vec{P}\right)\neq P_{\mathrm{Sdev}.}\left(\vec{X}|\overset{\leftarrow }{P}\right)$$
  An exception is the case of statistical meanic mirroring
  $$KC_{\mathrm{Smnp}\mathrm{rox}.}\left(\overset{\leftarrow }{X}|\vec{P}\right)=KC_{\mathrm{Smnp}\mathrm{rox}.}\left(\vec{X}|\overset{\leftarrow }{P}\right)$$
  $$P_{\mathrm{Smnp}\mathrm{rox}.}\left(\overset{\leftarrow }{X}|\vec{P}\right)=P_{\mathrm{Smnp}\mathrm{rox}.}\left(\vec{X}|\overset{\leftarrow }{P}\right)$$
  $$P_{\mathrm{Smnd}\mathrm{ev}.}\left(\overset{\leftarrow }{X}|\vec{P}\right)=P_{\mathrm{Smnd}\mathrm{ev}.}\left(\vec{X}|\overset{\leftarrow }{P}\right)$$
  where X,P∈R.
x. It is variant to a set duplication of a univariate dataset, except for statistical meanic mirroring, and the set duplication-invariance is effective to $P_{Smnprox.}$ and $P_{Smndev.}$, and not to $KC_{Smnprox.}$.
  Supposed we have a c duplicates a variable$\;X=(x_{1},x_{2},x_{3},.\ldots .,\;x_{n})$ and its statistical mirror $P=(p_{1},p_{2},p_{3},.\ldots .,p_{n})$, where P is the amplified estimate or reference value of the transformed variable X.
  $$KC_{Sprox.}\left(X,P\right)\neq KC_{Sprox.}\left(\left[X\right]*c,P\right)$$
  $$P_{Sprox.}\left(X,P\right)\neq P_{Sprox.}\left(\left[X\right]*c,P\right)$$
  $$P_{Sdev.}\left(X,P\right)\neq P_{Sdev.}\left(\left[X\right]*c,P\right)$$
  An exception is the case of statistical meanic mirroring
  $$KC_{Smnprox.}\left(X,P\right)\neq KC_{Smnprox.}\left(\left[X\right]*c,P\right)$$
  $$P_{Smnprox.}\left(X,P\right)=P_{Smnprox.}\left(\left[X\right]*c,P\right)$$
  $$P_{Smndev.}\left(X,P\right)=P_{Smndev.}\left(\left[X\right]*c,P\right)$$
  where X,P∈R;c∈N

### Python implementation

The proposed method of statistical mirroring, computing code was written in Python language. Get the Python code at Abdullahi [14] or via this link: https://data.mendeley.com/datasets/ppfvc65m2v/3.

Similarly, computer applications were developed to enhance accessibility and usability for users. Two applications were implemented and compressed into one folder. The first application, named “StatisticalMirroringApp1.2” enables users to directly input a single dataset and view the results within the application. The second application, “StatisticalMirroringApp2.2” allows users to upload one or multiple datasets in CSV or Excel format, processes the data, and enables the saving of outcomes as a new CSV file. You can access these applications through Abdullahi [32] or via this link: https://data.mendeley.com/datasets/gzkkg2p68t/2.

Here is the presentation of the implemented code for statistical mirroring.

### Statistical mirroring

*Meta-data:*

• Project: Kabirian-based Optinalysis
• Language: Python
• Libraries Used: NumPy, Statistics
• Other Python code(s) Used: Isomorphic optinalysis
• Functionality: Performs statistical dispersion estimation based on input data and parameters
• Author: Kabir Bindawa Abdullahi
• Version: 1.1

**Code Description:**

*Importing Libraries and Code:*

• The code begins by importing the necessary libraries, namely NumPy and Statistics.
• The Python code for Kabirian-based isomorphic optinalysis.

*Function Definitions:*

1. *statistical_mirroring:* The main function for statistical dispersion estimations.
  • *Input:* It takes an instruction_list, which is expected to contain a list of 6 elements:
    ○ *Data:* List of numerical values from a set of real numbers.
    ○ Mirror_principal_value: Numerical value for the design of a specific statistical mirror. It can be inputted as the mean, median, mode, maximum, minimum, or a reference numerical value or operation. This input partly determines the type of statistical mirroring to be performed.
    ○ Centering: Location removal from the dataset, customizable with or without returning the absolute positive or negative values. Centering can be commanded by the mean, median, mode, maximum, minimum, or a reference numerical value or operation.
    ○ Ordering: Theoretical arrangement of the dataset, customizable in either ascending or descending order.
    ○ Pairing: The type of isoreflective mapping, customizable as either Head-to-head or Tail-to-tail.
    ○ Print_result: Specifies which type of result(s) to print.
2. *preprocessing:* A nested function for preprocessing the input data. It uses the input commands in the main function, the statistical_mirroring, to perform data centering and ordering based on the input commands.
3. *isomorphic_optinalysis:* An imported and associated function not nested here. It calculates the Kabirian coefficient (*kc*), percentage similarity (*psim*), percentage dissimilarity (*pdsim*), and other alternate coefficients (i.e., *kcalt, kcalt1, kcalt2*) estimates using the transformed input data and the designed statistical mirror, based on the input pairing type. An Isomorphic optinalysis is established by Abdullahi [9] and its Python code is downloadable at Abdullahi [13].

*Main Process:*

• Input data and other parameters are extracted from the instruction_list.
• Preprocessing transformations and customizations of the data, as well as the design of a statistical mirror based on the input instructions.
• The Kabirian-based isomorphic optinalysis is computed and various estimates are calculated, including *kc, psim, pdsim, kc_alt1, kc_alt2,* and *kc_alt*.

*Output:*

• The output result depends on the print_result parameter in the instruction_list. The following options are available:
  ○ *“print:kc”:* Prints the Kabirian coefficient (*kc*).
  ○ *“print:psim”:* Prints the probability (percentage) of proximity (*pprox*).
  ○ *“print:pdsim”:* Prints the probability (percentage) of deviation (*pdev*).
  ○ *“print:kcalt1”:* Prints the A-alternative Kabirian coefficient_1 (*kcalt1*).
  ○ *“print:kcalt2”:* Prints the D-alternative Kabirian coefficient_2 (*kcalt2*).
  ○ *“print:all”:* Prints all estimates encapsulated in the all_estimates dictionary.

*Error Handling*

• If the input parameter is invalid, an error message is returned.

*How It's Used for Analysis:*

i. *Statistical mirroring:* This code estimates the statistical dispersion (such as coefficient, proximity, and deviation) of a dataset from a defined location estimate (such as mean, median, mode, maximum, minimum, or reference value).
ii. *Customizable input:* Users can choose which analysis to compute using different combinations of suitable parameters based on the task at hand.
iii. *Customizable output:* Users can choose which result(s) to print based on the outcome(s) of interest.
iv. *Data transformation:* The code may be useful for a certain spectrum of statistical data analyses and further transformations on a dataset.
v. *Extensibility:* It can be extended to support additional analyses in multivariate settings.
vi. Overall, this code provides an advanced and comprehensive tool for statistical measure of dispersion, with flexibility in the input parameters and selecting which outcome(s) to display.

### Drawbacks and limitations of statistical mirroring

The following are some of the identified drawbacks and limitations of statistical mirroring:

i. The given random ordering of elements of the list of the variable(s) is not preserved, thus an efficient theoretical ordering (i.e., ascend or descend order) is used and ensures permutation-invariance. Therefore, it is not suitable where a pattern of the variables is important.
ii. A suitable and efficient pairing style or alternate reflection has to be chosen and adopted for repeatability and comparison of results. This excludes only statistical meanic mirroring.
iii. The two possible Kabirian bi-coefficients do not function on the same optinalytic scale. For comparison of results, estimates with the mixed Kabirian coefficients should either be translated forward or otherwise uniformed by backward alternate translation.
iv. Where location shift transformation of a variable is a specific concern for a location shift-invariance estimator, statistical mirroring is not a suitable alternative.

### Manual calculations in statistical mirroring

Table 2 demonstrates a manual calculation on a sample dataset using the proposed statistical meanic mirroring method.

**Table 2:** Manual demonstration of statistical mirroring using a sample dataset.

Parameters and operations	Selection	Statistical absolute meanic mirroring: steps and calculations	
Random observations	Values of data X	Data: X=[−0.02,6.31,6.20,−4.36,1.22,2.87]	
Optional data centering	By the mean of data Xand return absolute positive values	Data: $t_{c^{+}}\left(X\right)=\left[2.06,\;4.27,\;4.16,\;6.40,\;0.82,\;0.83\right]$	
Compulsory data ordering	Ascending order	Data: $t_{c^{+}\to o}\left(X\right)=\left[0.82,\;0.83,\;2.06,\;4.16,\;4.27,\;6.40\right]$	
Statistical meanic mirror design	The principal value is the mean of the transformed variable $t_{c^{+}\to o}\left(X\right)$.	$P=\left[p\right]*n=\left(p_{1},p_{2},p_{3},.\ldots .,p_{n}\right)$ Such that the principal value is the p=g(X), p∈P, P is the statistical mirror, n∈N, X∈R, and the g function is the defined location estimates. Meanic mirror: P=[3.09]*6=[3.09,3.09,3.09,3.09,3.09,3.09]	
Optinalytic construction and pairing style	Head-to-head (H—H) pairing is used but can be chosen as tail-to-tail (T-T) pairing, of the isoreflective pairs	Such that δ∉X,P;X,P,δ∈R; $R\subseteq \{r\in R^{+*}|r=n*k,\;n\in N,\;k>0\}$ or alternatively $R\subseteq \{r\in R^{-*}|r=-n*k,\;n\in N,\;k>0\}$; n∈N; and X&P are isoreflective pair on a chosen pairing about a central point δ.	
Normalization of δ and data substitution	By default, normalization is zero, δ=0		
Proceeding to Kabirian-based isomorphic optinalysis [9]			
Calculations/Computations [9]	Numeration (the sum of variables × median optiscale)	$r_{n+1}\left[\sum_{i=1}^{n}\left(x_{i}+\delta +p_{i}\right)\right]=259.56$	
	Denomination (the sum of the scalements)	$\sum_{i=1}^{n}\sum_{j=n+2}^{2n+1}\left(r_{i}x_{i}+r_{n+1}\delta +r_{j}p_{i}\right)=279.72$	
Kabirian coefficient and optinalysis-to-probability translations [9]	Kabirian coefficient of statistical proximity	$KC_{Sprox.\;}\left(X,P\right)=\frac{r_{n+1}\left[\sum_{i=1}^{n}\left(x_{i}+\delta +p_{i}\right)\right]}{\sum_{i=1}^{n}\sum_{j=n+2}^{2n+1}\left(r_{i}x_{i}+r_{n+1}\delta +r_{j}p_{i}\right)}=0.927928$	
	Estimate of statistical proximity	$\forall \;0\leq K_{c}\leq 1$	$P_{Sprox.}\left(X,P\right)=\frac{\left(nr_{1}+r_{1}\right)-K_{c}\left(2nr_{1}+r_{1}\right)}{r_{1}\times K_{c}-\left(nr_{1}+r_{1}\right)}=0.8338\;=83.38\%$
		$\forall \;1\leq K_{c}\leq n+1;$ $K_{c}\geq n+1;$ $\&\;K_{c}\leq 0$	$P_{Sprox.}\left(X,P\right)=\frac{\left(nr_{1}+r_{1}\right)-r_{1}K_{c}}{\left(2nr_{1}+r_{1}\right)K_{c}-\left(nr_{1}+r_{1}\right)}$
	Estimate of statistical deviation	$\forall \;P_{Sprox.}\geq 0$	$P_{Sdev.\;}\left(X,P\right)=1-P_{Sprox.}\left(X,P\right)=0.1662=16.62\%$
		$\forall \;P_{Sproc.}\leq 0$	$P_{Sdev.\;}\left(X,P\right)=-1-P_{Sprox.}\left(X,P\right)$

## Method application, validation, and comparison

### Methods comparison using artificial datasets

In this section, attention was focused on the application, validation, and comparison of the proposed statistical mirroring estimators against established reference estimators. Due to the methodological broadness in types of statistical mirroring, the limitation is restricted to only one of the approaches, the statistical meanic mirroring. The primary focus is on the absolute and raw statistical meanic mirroring (using ascending order, and head-to-head pairing as the selected parameter option), as described earlier; and some of the well-established reference estimators, namely the standard deviation and coefficient of variation. To rigorously evaluate their performance, a Monte Carlo simulation experiment was conducted using artificial datasets, predominantly generated from normal Gaussian and Gaussian mixture model distributions because they have the desired parameters to manipulate and describe a dispersion of a univariate random variable.

#### Description of estimators

Table 3 provides an overview of the estimators used in the study, along with their respective acronyms and method status. The proposed estimators include the Kabirian coefficient of statistical absolute or raw meanic proximity (*Kc*), Probability of statistical absolute or raw meanic proximity (*Pprox*), Probability of statistical absolute or raw meanic deviation (*Pdev*), A-alternate Kabirian coefficient of statistical absolute or raw meanic proximity (*Kcalt1*), and D-alternate Kabirian coefficient of statistical absolute or raw meanic proximity (*Kcalt2*). The comparison also involves classical estimators like the standard deviation (*Std*) and coefficient of variation (CV).

**Table 3:** Description of the estimators and the acronyms used to represent them.

Methods	Description of the acronyms	Method status
*Std*	Standard deviation	Classical
*CV*	Coefficient of variation	Classical
*Kc*	Kabirian coefficient of absolute or raw meanic proximity	Proposed
*Pprox*	Probability of absolute or raw meanic proximity	Proposed
*Pdev*	Probability of absolute or raw meanic deviation	Proposed
*Kcalt1*	A-alternate Kabirian coefficient of absolute or raw meanic proximity	Proposed
*Kcalt2*	D-alternate Kabirian coefficient of absolute or raw meanic proximity	Proposed

#### Artificial datasets generation

To explore various combinations of parameters and generate a dataset for analysis, we utilized a Python command that efficiently produces tuples of values. This approach allows us to systematically pair elements from different lists and incorporate additional fixed values. Monte Carlo simulation was employed to generate artificial datasets, predominantly from normal (N[μ,σ,N,S]) and Gaussian mixture model ($GM[\left[\mu_{1},\mu_{2}\right],\left[\sigma_{1},\sigma_{2}\right],p,\;N,\;S]$) distributions.

**Fig. 2:**
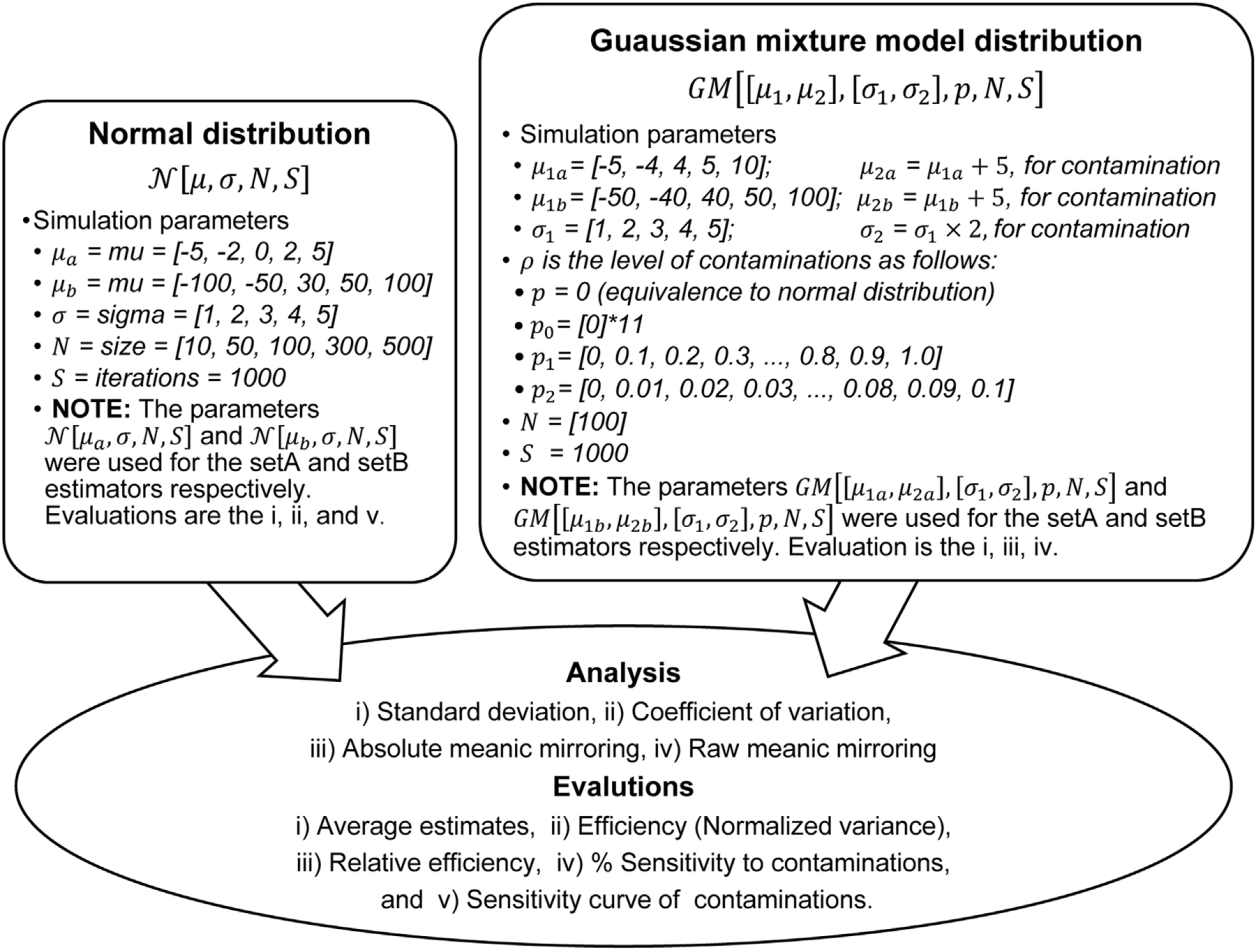
Parameter presentation and simulation design for data generation, analysis, and evaluation of dispersion estimators under normal and Gaussian mixture model distributions.
ii. 125,000 datasets were generated for various systematic combinations of a set of sample sizes (N), a set of location parameters (μ), and a set of shape parameters (σ), over several iterations (S). For each combination of x from N, y from μ, and z from σ, a tuple (x, y, z, iteration) was formed and added to the list datasets. The resulting list contained all the 125 possible combinations of the elements in N, μ, and σ with the corresponding value of iteration in each tuple. For more details, visit the simulation process and the Python codes at Abdullahi [15].
iii. Step (ii) was followed for the first set of estimators, and 125,000 datasets were generated.
iv. Step (ii) was followed for the second set of estimators, and another 125,000 datasets were generated.
v. Overall, a total of 250,000 datasets were generated all together.

1. Normal Distribution (N):

The simulation follows these steps:

i. Fig. 2 presented the outline of the simulation design and parameters for the generation of datasets from a univariate random normal distribution.

2. *Gaussian Mixture Model Distribution (* GM *):*
  ➢ Two scenarios of simulations were considered a) normal, and b) alternating Gaussian mixture model distributions. It is equivalent to a normal distribution for ρ=0.
    • Two sets of estimators were considered: a) set A estimators, consisting of standard deviation and absolute meanic mirroring; b) set B estimators, consisting of coefficient of variation and raw meanic mirroring.
      ○ For each set of estimators, two scenarios of contamination types were considered: a) location shift contaminations and b) scaling contaminations.
        ✓ For each contamination type, two scenarios of contamination levels were considered: a) higher contamination levels ($p_{1}$) to study estimators' sensitivity curve to location shift and scaling, and b) lower contamination levels ($p_{2}$) for a comparative prediction of contamination resistance.

The simulation follows these steps:

i. Fig. 2 presented the outline of the simulation design and parameters for the generation of datasets from a univariate random Gaussian mixture model distribution.
ii. 275,000 datasets were generated for various systematic combinations of a set of contamination levels ($p_{x}$), a set of location mixture parameters ($\left[\mu_{1},\mu_{2}\right]$), a set of shape mixture parameters ($\left[\sigma_{1},\sigma_{2}\right]$), and a set of sample sizes (N), over several iterations (S). For each combination of c from $p_{x}$, ‘*x* from N, *y* from $\left[\mu_{1},\mu_{2}\right]$, and *z* from $\left[\sigma_{1},\sigma_{2}\right]$, a tuple (*c, x, y, z, iteration*) was formed and added to the list datasets. The resulting list contained all 275 possible combinations of the elements in $p_{x}$, N, $\left[\mu_{1},\mu_{2}\right]$, and $\left[\sigma_{1},\sigma_{2}\right]$ with the corresponding value of iteration in each tuple. For more details, visit the simulation process and the Python codes at Abdullahi [15].
iii. The step (ii) was repeated with zero contaminations ($p_{0}$) of the corresponding sets from mixture datasets and 275,000 normal datasets were generated.
iv. The steps (ii) – (iii) were followed, and 4 simulations were run to test the sensitivity of the estimators to location shift contaminations, as follows:
  a. The first and second sets of estimators under the first ($p_{1}$) set of contamination levels, and 1,100,000 datasets were generated.
  b. The first and second sets of estimators under the second ($p_{2}$) set of contamination levels, and 1,100,000 datasets were generated.
v. The steps (ii) – (iii) were followed, and 4 simulations were run to test the sensitivity of the estimators to scaling contaminations, as follows:
  a. The first and second sets of estimators under the first ($p_{1}$) set of contamination levels, and 1,100,000 datasets were generated.
  b. The first and second sets of estimators under the second ($p_{2}$) set of contamination levels, and 1,100,000 datasets were generated.
vi. Overall, a total of 4,400,000 datasets were generated altogether.

#### Data analysis and comparison

During data analysis, two sets of estimators were identified for direct comparisons:

*Set A Estimators:*

• Standard deviation (*Std*): Location-invariant estimator.
• Absolute meanic mirroring: Scaloc-invariant estimator.

*Set B Estimators:*

• Coefficient of variation (*CV*): Scale-invariant estimator.
• Raw meanic mirroring: Scale-invariant estimator.

Python codes were utilized for the complete simulation process, data analysis, and evaluation of the dispersion estimators, ensuring reproducibility of the results. The source code is available in a Mendeley Data repository, documented and deposited by Abdullahi [15].

### Estimates and performance evaluation of the estimators

In this section, we delve into the estimation and performance evaluation of the proposed statistical mirroring estimators, comparing them with established reference estimators such as standard deviation and coefficient of variation. The evaluation metrics include average estimates, efficiency, and relative efficiency, sensitivity to contamination, sensitivity curves, and statistical analysis. The analyses were conducted using Python scripts implemented by Abdullahi [15].

#### Estimation procedure

The estimation process involved calculating the population standard deviation and coefficient of variation using Python functions. Additionally, the proximity and deviation estimates of the proposed statistical mirroring method were evaluated using Python codes implemented by Abdullahi [13] and Abdullahi [14]. To ensure comparability, a Min-Max scaler was applied for normalization before efficiency and relative efficiency evaluation.

#### Dispersion estimates

The dispersion estimates of the reference estimators were computed using a population standard deviation (Eq. (9)) and then the coefficient of variation (Eq. (10)), while the dispersion estimates of the proposed estimators (i.e., the absolute and raw statistical meanic mirroring) were computed as described earlier.

(10) $$Standard\;deviation\;\left(\beta \right)=\sqrt{\frac{1}{N}\sum_{i=1}^{N}{\left(X_{i}-\bar{X}\right)}^{2}}$$

(11) $$Coefficient\;of\;variation\;\left(\beta \right)=\frac{\sqrt{\frac{1}{N}\sum_{i=1}^{N}{\left(X_{i}-\bar{X}\right)}^{2}}}{\bar{X}}$$

Where β is the dispersion estimates of any estimator, N is the number of values (sample size) for a variable, $X_{i}$ is the ith value of the variable, $\bar{X}$ is the average of X distribution.

#### Evaluation metrics

The evaluation of the estimators based on the estimates obtained was as follows:

i. *Average estimates of the estimators:* The estimates β can be negative or positive with a coefficient of variation, and the influence of this variation was removed by taking the absolute of the β estimates. The average of the dispersion estimates β was expressed by the total iterations performed (Eq. (11)).
  (12) $$Average\;estimate=\;\frac{1}{S}\sum_{i=1}^{S}\beta_{i}$$
ii. *Efficiency and relative efficiency*: Concerning efficiency (precision of the estimates), variance (Eq. (13)) was calculated. The variance is connected to the variability of the estimated values, and little variation specifies that the estimator is efficient or precise [16]. To achieve reliable comparability among the estimators, the estimates were first normalized by a Min-Max scalar (Eq. (12)) to remove all location and scale influences [17,18]. The relative efficiency (RE) (Eq. (14)) was calculated to compare the proposed estimators relative to the classical one. If: RE=1, both the reference and proposed estimators are equally efficient; RE<1, the proposed estimator is more efficient than the reference estimator; RE>1, the proposed estimator is less efficient than the reference estimator.
  (13) $$MinMax\_normalized\;estimate\;\left(\omega \right)=\frac{\beta_{i}-\beta_{min}}{\beta_{max}-\beta_{min}}$$
  (14) $$Efficiency\;\left(Eff\right)=Variance\;of\;\omega =\frac{1}{S}\sum_{i=1}^{S}{\left(\omega_{i}-\bar{\omega }\right)}^{2}$$
  (15) $$Relative\;efficiency\;\left(RE\right)=\frac{Eff_{re}}{Eff_{pe}}$$

where $\beta_{min}$ is the minimum value of the β estimate; $\beta_{max}$ is the maximum value of the β estimate; $\bar{\omega }=\sum_{i=1}^{S}\omega_{i}/S$; $\omega_{i}$ is the estimate of interest within each of the i=1,……S simulations; $Eff_{re}$ is the efficiency of the reference(gold standard) estimator; $Eff_{pe}$ is the efficiency of the proposed estimator.

i. *Sensitivity to contamination:* Concerning the sensitivity (measured as the relative absolute error) of the estimator to contamination, the absolute deviance between the contaminated estimate and the uncontaminated estimate was used. The sensitivity has been examined in relative terms to be comparable. The Eq. (15) quantifies the percentage sensitivity to a contamination.
  (16) $$\%\;sensitivity\;to\;contamination=\;\left[\frac{\left|\bar{\hat{\beta }}-\bar{\beta }\right|}{\bar{\beta }}\right]\times 100$$

where $\bar{\beta }=\sum_{i=1}^{S}\beta_{i}/S$ is the value of the normal uncontaminated estimate; $\bar{\hat{\beta }}=\sum_{i=1}^{S}{\hat{\beta }}_{i}/S$ is the value of the contaminated estimate; and S is the number of iterations of the simulation performed; and $\beta_{i}$ and ${\hat{\beta }}_{i}$ are the estimates of interest within each of the i=1,……S simulations.

i. *Sensitivity curve:* The mean of the percentage sensitivity (i.e., mean relative absolute error) for all the different parameters (i.e., mean and sigma at a given fixed sample size) combinations was plotted against the different levels of contaminations. The plot expressed the sensitivity curve of the estimator and was used to model and evaluate the level of contaminations that an estimator can resist 0.05 (5%) sensitivity by location shift and scaling.
ii. *Statistical analysis:* Two-tailed unpaired student *t*-test was used to compare the statistical significance of the differences between the performance (specifically the efficiency) estimates of two directly comparing set A and set B dispersion estimators.
iii. *The best estimator:* The best dispersion estimator is one with a bounded range of dispersion estimates, invariance (robust) to both scale and location shift, lowest efficiency or relative efficiency, and lowest sensitivity to contaminations (resistant to data contaminations) [11]. These properties, representatives of the most basic essential requirements, do not exhaustively address all the desirable properties of good estimators of dispersion, but other properties such as sufficiency are also important.

### Methods comparison under temperature measurement scales

Temperature measurement scales are diverse, serving various purposes in daily life and scientific research. Developing an estimator robust to temperature scale variations is a challenging yet promising endeavor. Such an estimator could promote consistency, comparability, and integration across different temperature scales, fostering advancements in diverse fields and applications.

These temperature scales are derived from interval or ratio measurement scales. The key distinction between the two categories lies in whether or not there is an absolute zero point. Kelvin, Rankine, and Delisle have a true zero point (absolute zero), making them ratio scales. On the other hand, Degree Celsius, Fahrenheit, Romer, Newton, and Reaumur lack a true zero point, so they are considered interval scales.

Let x be a temperature measurement in Degree Celsius unit ( °C). The temperature equivalences in other temperature scales are provided by Eqs. (8)–(14).

(17) Kelvin=x+273.15

(18) $$Rankine=\frac{\left(x+273.15\right)\times 9}{5}$$

(19) $$Fahrenheit=\frac{x\times 9}{5}+32$$

(20) $$Romer=\frac{x\times 21}{40}+7.5$$

(21) $$Newton=\frac{x\times 33}{100}$$

(22) $$Reaumur=\frac{x\times 4}{5}$$

(23) $$Delisle=\frac{x\times 9}{5}+32$$

The best estimator of dispersion gives the same dispersion estimates (i.e., zero sensitivity) for whatever temperature scale is used.

A sample of real-life temperature measurements was used to demonstrate the robustness of the dispersion estimators. The data was collected from https://en.climate-data.org/africa/nigeria/katsina-355/r/january-1/, retrieved in December 2023. The data in Table 4 is a collection of thirty (30) years (from 1991 – 2021) average of monthly temperature of Katsina State, Nigeria.

**Table 4:** Thirty (30) years average of monthly temperature (°C) of Katsina State, Nigeria.

Month	Jan.	Feb.	Mar.	Apr.	May	Jun.	Jul.	Aug.	Sep.	Oct.	Nov.	Dec.
**Avg.** °**C**	21.6	24.7	28.3	31.2	31.5	29.7	26.9	25.4	26.7	27.7	25.7	22.2

## Results

### Transformation invariance

**Fig. 3:**
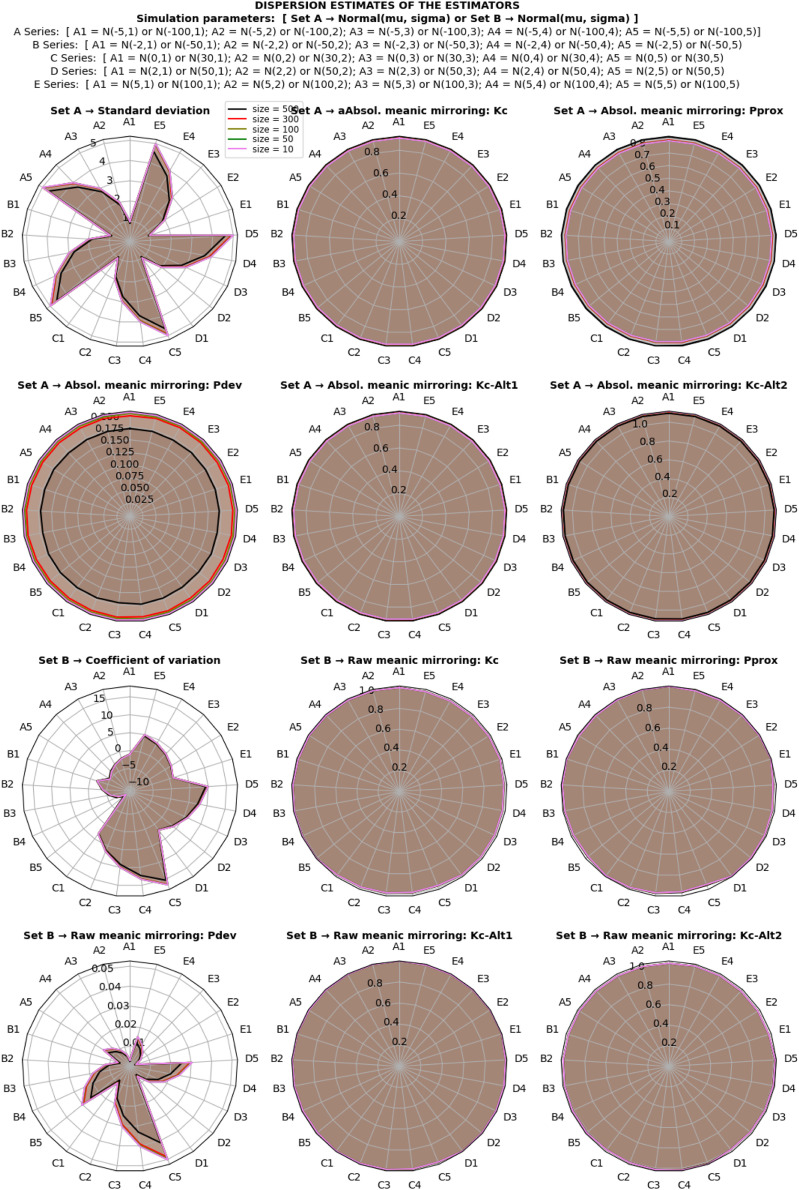
Dispersion estimates of the estimators under normal distribution.

The study investigated the behavior of dispersion estimates under a normal distribution using both classical and proposed estimation methods. The simulation involved various scale and location shift transformations under different sample sizes, as depicted in Fig. 3. The assessment of estimate behavior focused on invariance characteristics, categorizing estimators as location-variance, scale-invariance, or scaloc-invariance based on their resistance to specific transformations.

The results, illustrated in Fig. 3, revealed that the standard deviation is a location-invariant estimator, showing resilience against location shift effects. In contrast, the coefficient of variation and raw meanic deviation demonstrated scale-invariance, resisting transformations induced by scaling effects. Notably, the absolute meanic deviation emerged as a scaloc-invariant estimator, robust against both location shift and scaling effects. Each estimator maintained its characteristic invariance even as sample sizes increased, converging consistently toward true population estimates.

However, the estimator of relative dispersion, represented by the coefficient of variation, exhibited sensitivity to the direction of the mean. This sensitivity resulted in a range of positive and negative integers, depending on the mean's direction in the distribution. In contrast, the proposed estimators showcased stability, eliminating reliance on the directionality of the mean (See Fig. 3).

Further exploration of derivative estimators, including the Kabirian coefficient of meanic proximity, meanic proximity, and alternate Kabirian coefficient, demonstrated comparative scaloc-invariance and scale-invariance to transformations. This stood in partial opposition to the behaviors observed in the standard deviation and coefficient of variation.

Moreover, the standard deviation, while a location-invariant estimator, was unbounded, capable of assuming a range of non-negative values based on data variability. The coefficient of variation, a bounded estimator, exhibited limitations when the standard deviation surpassed the mean or approached zero. In contrast, proposed estimators, encompassing both absolute and raw meanic deviations, were bounded within a unified probabilistic range for all dispersion estimations, providing clearer and more predictable boundaries.

Similarly, derivative estimators, such as meanic proximity and the alternate Kabirian coefficient, presented a distinct advantage with a bounded range of estimates. These findings underscore the robustness, stability, and bounded probabilistic ranges of the proposed estimators, offering valuable insights into their behavior under diverse conditions and highlighting their potential applications in practical scenarios.

### Efficiency and relative efficiency

The efficiency and relative efficiency of dispersion estimates under normal distribution were systematically evaluated through the application of both classical and proposed estimation methods. Utilizing simulation parameters involving scale and location shift transformations across diverse sample sizes, as illustrated in Fig. 2, the assessment provided comprehensive insights into the performance of the estimators.

**Fig. 4:**
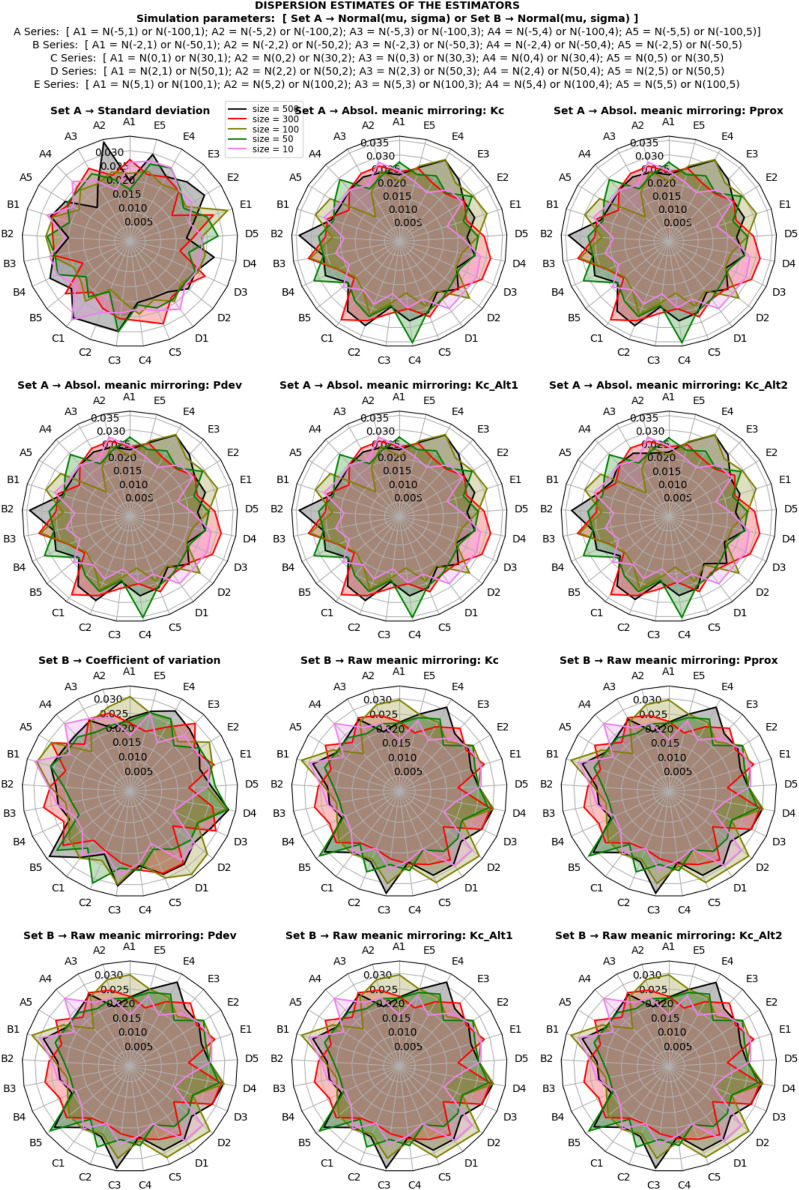
Efficiency of dispersion estimates for the estimators under normal distribution.

Fig. 4 depicts the efficiency evaluation results, revealing a consistent and indistinct pattern between the efficiencies of classical and proposed estimators under normal distribution. Notably, these efficiencies exhibited a stable response to simulation transformations, with a subtle variation observed as sample sizes increased.

**Fig. 5:**
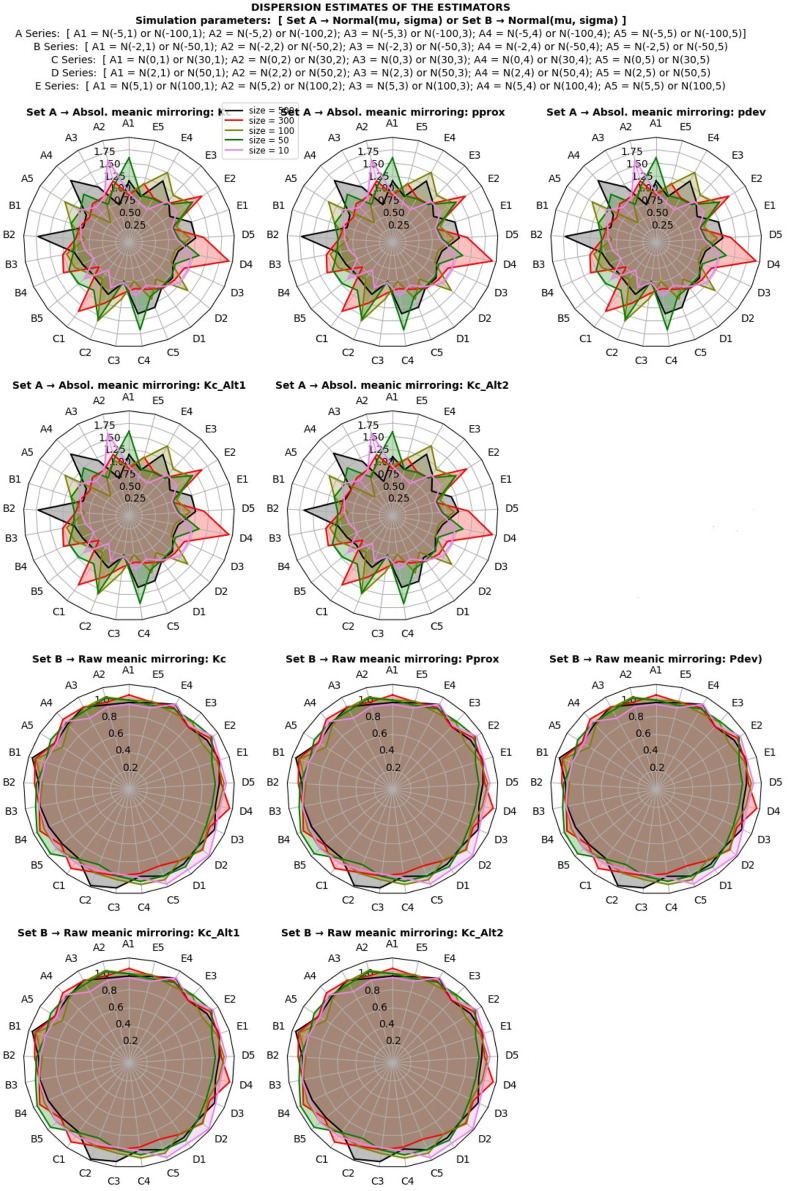
Relative efficiency of dispersion estimates for the estimators under normal distribution.

The findings in Fig. 5 showcased commendable relative efficiency (RE) characteristics for all estimators, including the standard deviation, absolute meanic deviation, raw meanic deviation, and coefficient of variation. The standard deviation, in particular, demonstrated higher efficiency and slightly outperformed (overall average RE = 1.08 ± 0.25 *Std*; 19.46*% Amd*) the absolute meanic deviation, with statistically significant differences (*P* < 0.05). However, the coefficient of variation and the raw meanic deviation showed asymptotically equal efficiency (overall average RE = 1.00 ± 0.05 *Std*; 17.89*% Amd*) and exhibited statistically no significant differences (*P* < 0.05). Note that the*% Amd* represents the percentage absolute meanic deviation of the statistical meanic mirroring estimates evaluated.

Additionally, derivative estimators, such as the Kabirian coefficient of meanic proximity, meanic proximity, and the alternate Kabirian coefficient, demonstrated comparable efficiencies (precision) when applied to random samples from a symmetrical normal distribution.

### Sensitivity to location shift contaminations

**Fig. 6:**
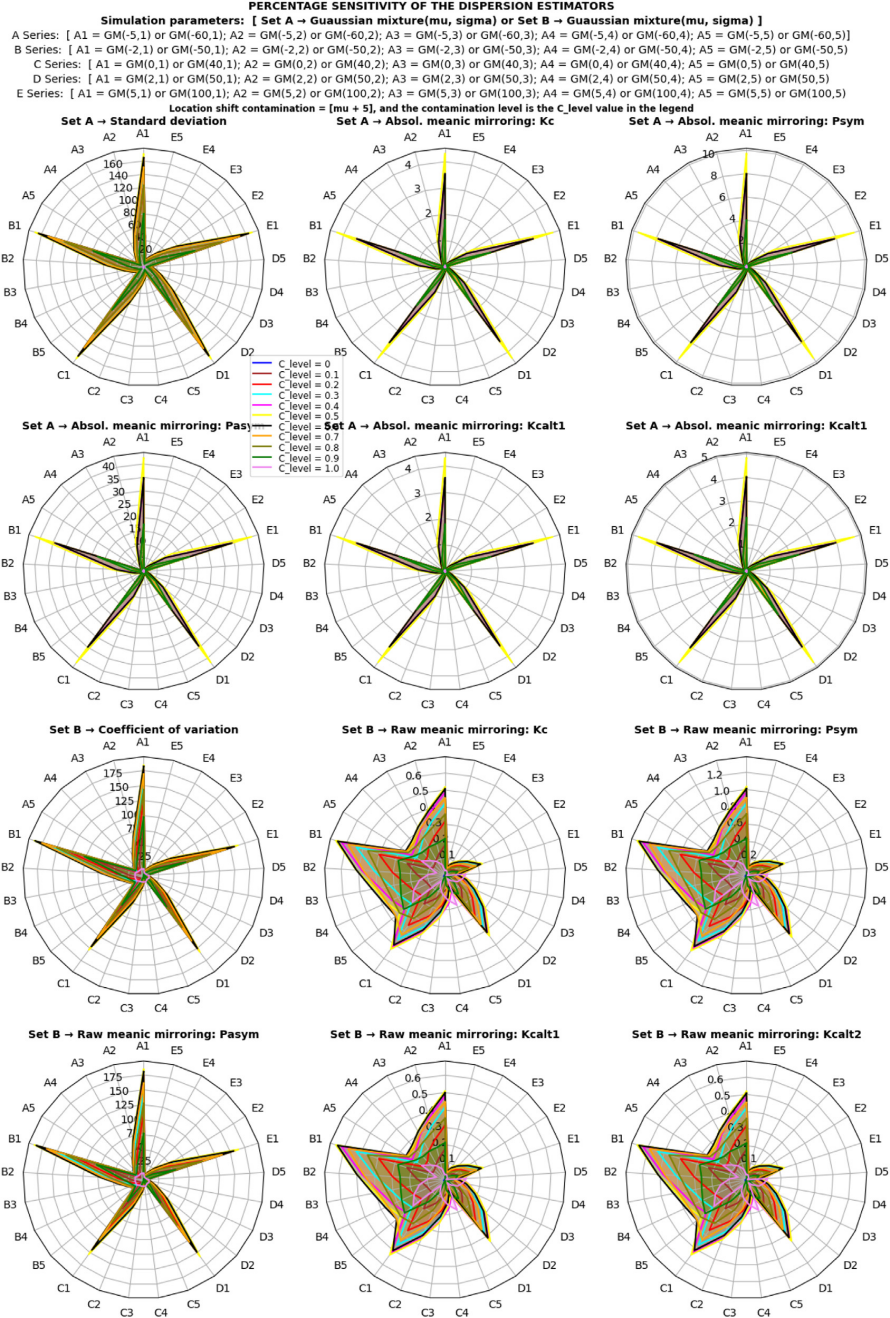
Sensitivity percentage of dispersion estimates for the estimators under the Gaussian mixture model with location shift contaminations.

**Fig. 7:**
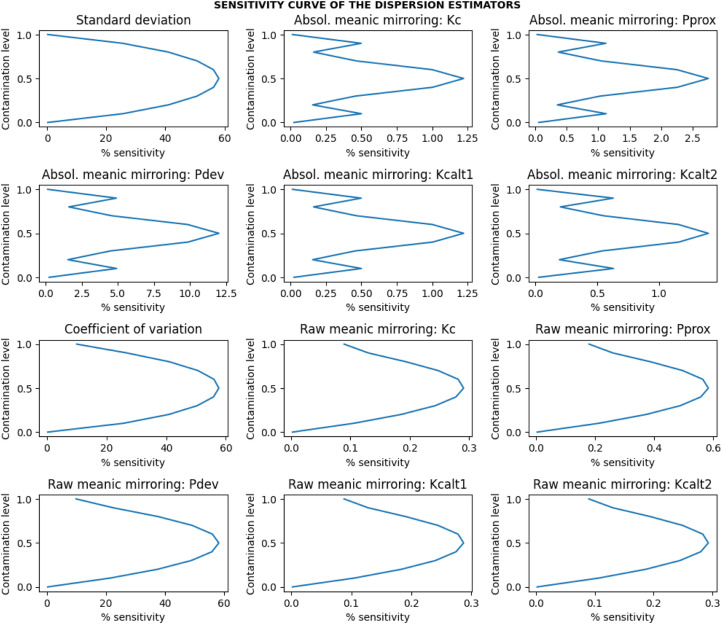
Sensitivity curve of dispersion estimates for the estimators under Gaussian mixture model with location shift contaminations.

**Fig. A1:**
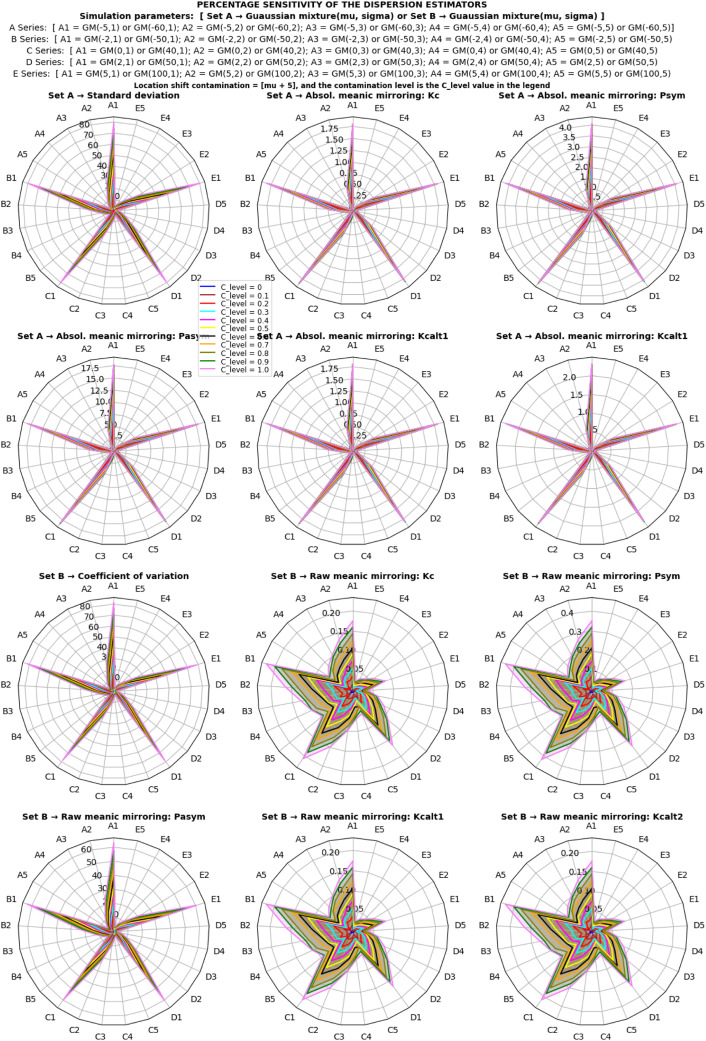
Sensitivity percentage of dispersion estimates for the estimators under Gaussian mixture model with location shift contaminations.

**Fig. A2:**
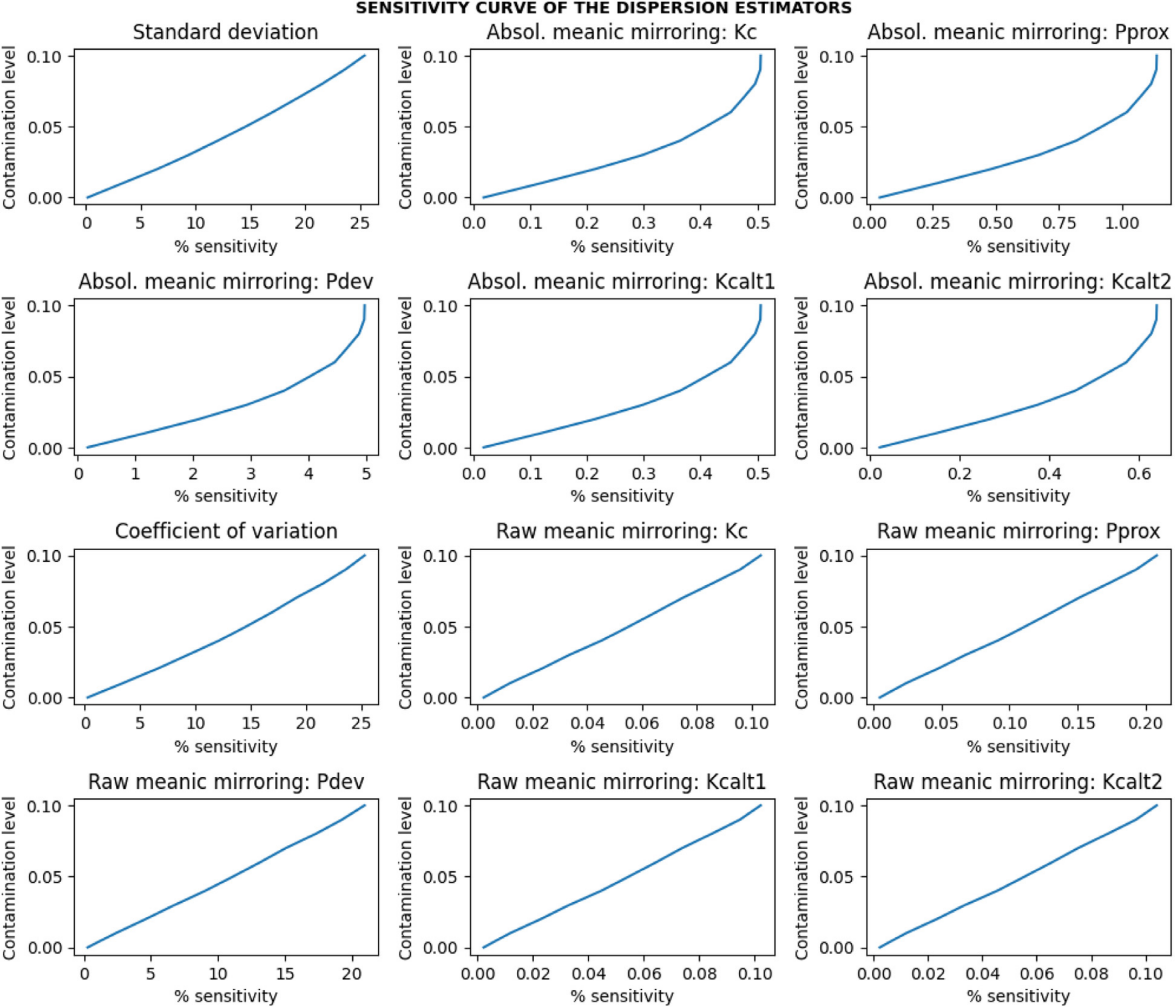
Sensitivity curve of dispersion estimates for the estimators under Gaussian mixture model with location shift contaminations.

The sensitivity to contamination of dispersion estimates from both classical and proposed estimators under Gaussian mixture model distributions was scrutinized using the simulation parameters outlined in Fig. 2. The outcomes of this sensitivity evaluation are depicted in Fig. 6, Fig. 7 and Figs. A1–A2 of Appendix A.

Notably, the absolute meanic deviation exhibited lower sensitivity to contaminations induced by location shift compared to the standard deviation. Similarly, the raw meanic deviation displayed exhibited almost similar sensitivity compared to the coefficient of variation (see Fig. 6).

Specifically, the absolute meanic deviation demonstrated resistance against a 7.90% level of location shift contaminations, maintaining a below 5% relative error. It notably exhibited a lower maximum sensitivity effect, forming a symmetrical curve around the 50% level of location shift contaminations. An impressive decrease in sensitivity was observed around the pericentre (equivalent to around the 25% level of location shift contaminations) of the isoreflective pair with absolute meanic mirroring. This sensitivity toward the pericentre is less pronounced than the sensitivity observed away from the pericentre. In contrast, the standard deviation could only resist a 1.50% level of location shift contaminations to achieve a below 5% relative error, showcasing a higher maximum sensitivity effect that formed a symmetrical curve around the 50% level of location shift contaminations (see Fig. 7, Fig. A1–A2 of Appendix A).

Furthermore, the raw meanic deviation showcased resistance against a 2.20% level of location shift contaminations, ensuring a below 5% relative error. It demonstrated a lower maximum sensitivity effect, forming a symmetrical curve around the 50% level of location shift contaminations. Conversely, the coefficient of variation could only resist a 1.50% level of location shift contaminations to maintain a below 5% relative error, with an almost equivalent lower maximum sensitivity effect that formed a symmetrical curve around the 50% level of location shift contaminations (see Fig. 7, Fig. A1–A2 of Appendix A).

Similarly, other derivative estimators of absolute and raw meanic mirroring, such as the Kabirian coefficient of meanic proximity, meanic proximity, and the alternate Kabirian coefficient, exhibited a more robust and resilient response to location shift contaminations when compared to the standard deviation and coefficient of variation (see Fig. 6, Fig. 7).

### Sensitivity to scaling contaminations

**Fig. 8:**
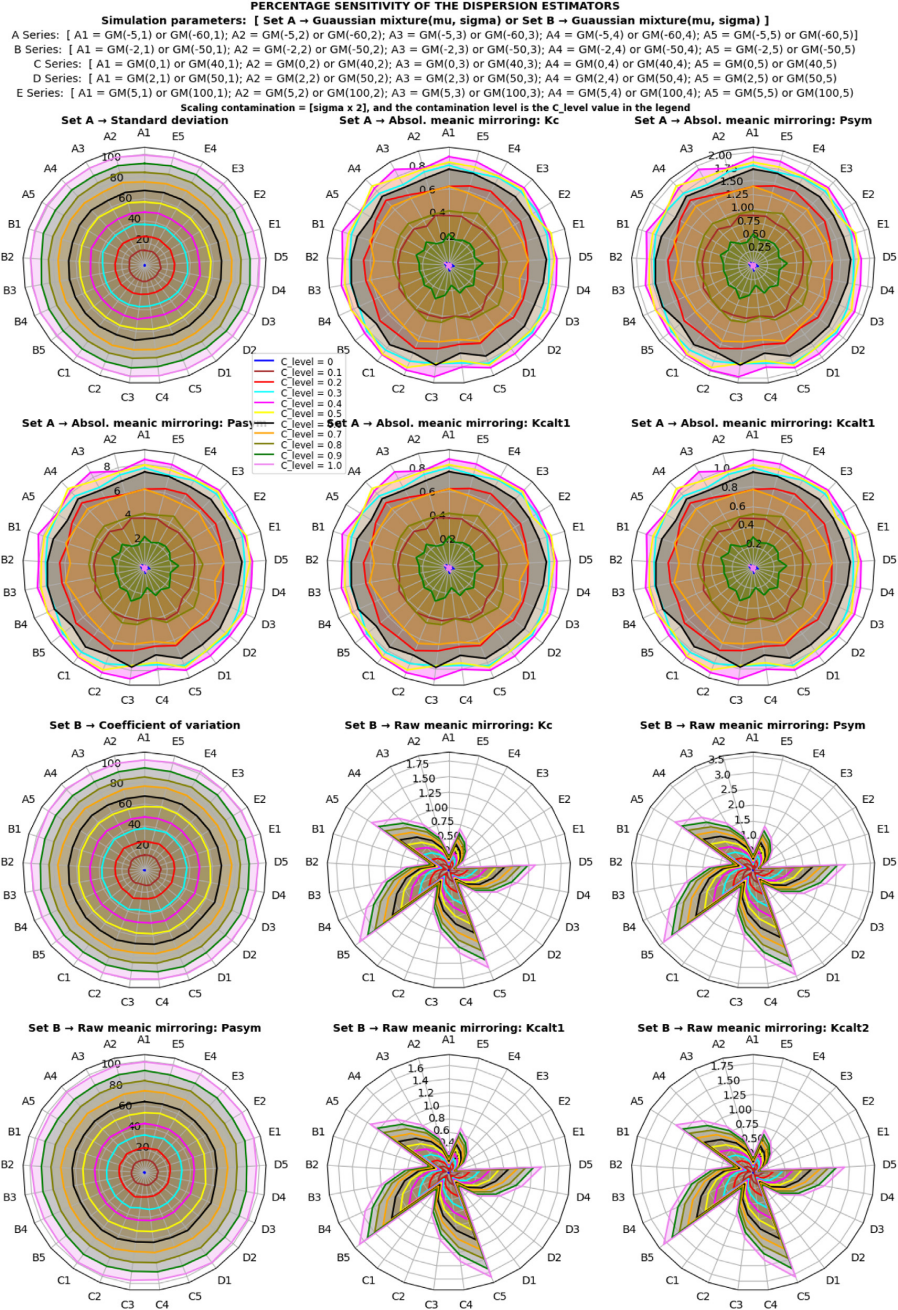
Sensitivity percentage of dispersion estimates for the estimators under the Gaussian mixture model with scaling contaminations.

**Fig. 9:**
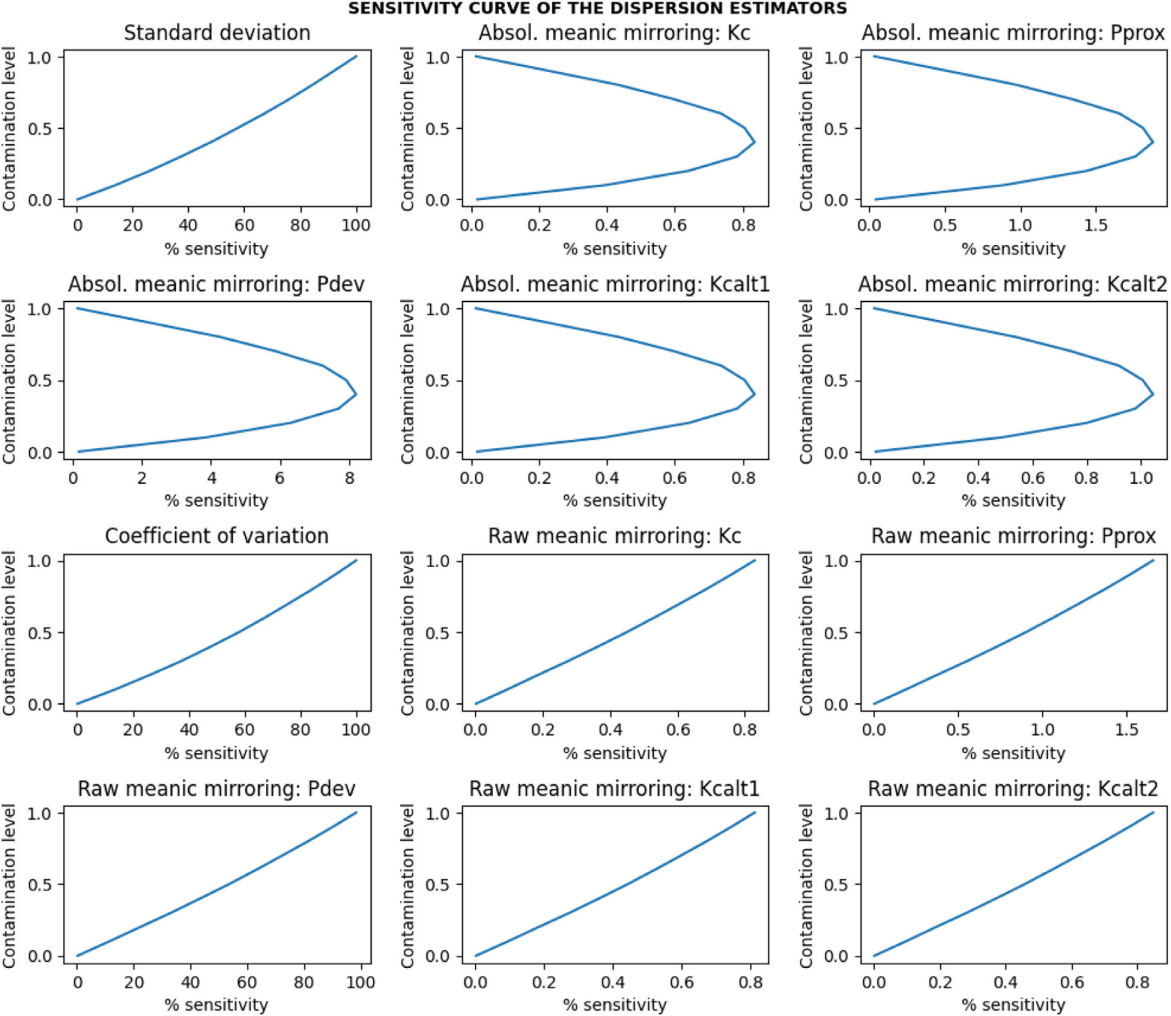
Sensitivity curve of dispersion estimates for the estimators under Gaussian mixture model with scaling contaminations.

The evaluation of sensitivity to contamination in dispersion estimates from both classical and proposed estimators under Gaussian mixture model distribution utilized the specified simulation parameters in Fig. 2. These findings are detailed in Fig. 8, Fig. 9 and Figs. A1–A2 of Appendix B.

Notably, the absolute meanic deviation demonstrated lower sensitivity to contaminations induced by scaling compared to the standard deviation. Similarly, the coefficient of variation exhibited lower sensitivity than the raw meanic deviation (see Fig. 8).

**Fig. B1:**
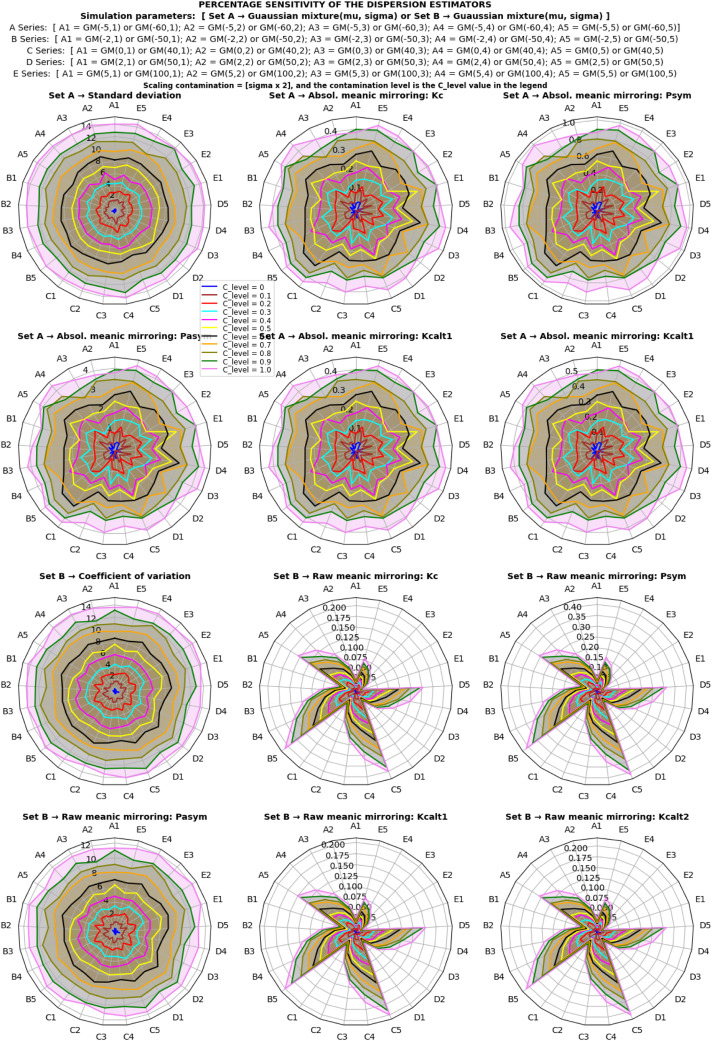
Sensitivity percentage of dispersion estimates for the estimators under Gaussian mixture model with scaling contaminations.

**Fig. B2:**
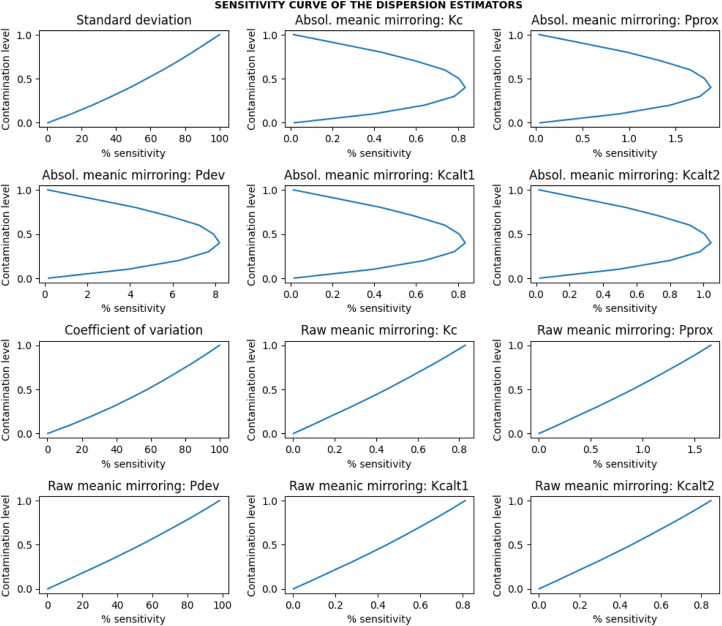
Sensitivity curve of dispersion estimates for the estimators under Gaussian mixture model with scaling contaminations.

Specifically, the absolute meanic deviation showcased resistance against a 12.40% level of scaling contaminations, maintaining a below 5% relative error. It demonstrated a lower maximum sensitivity effect, forming a symmetrical curve around the 50% level of scaling contaminations. In contrast, the standard deviation could only resist a 3.50% level of scaling contaminations to achieve a below 5% relative error, displaying a higher maximum sensitivity effect that grew polynomially as the level of scaling contaminations increased (see Fig. 9, Fig. B1–B2 of Appendix B).

Furthermore, the raw meanic deviation demonstrated resistance against a 3.50% level of scaling contaminations, ensuring a below 5% relative error. It displayed a lower maximum sensitivity effect, growing polynomially as the level of scaling contaminations increased. Conversely, the coefficient of variation could only resist a 4.30% level of scaling contaminations to maintain a below 5% relative error, with a higher maximum sensitivity effect that grew polynomially as the level of scaling contaminations increased (see Fig. 9, Fig. B1–B2 of Appendix B).

Similarly, other derivative estimators of absolute and raw meanic mirroring, such as the Kabirian coefficient of meanic proximity, meanic proximity, and the alternate Kabirian coefficient, exhibited more comparative robustness and resilience to scaling contaminations compared to the standard deviation and coefficient of variation (see Fig. 8, Fig. 9).

### Dispersion estimators under temperature measurement scales

The robustness of dispersion estimates, assessed across eight distinct temperature scales using both classical and proposed estimators based on real-life temperature measurements, provides insightful findings presented in Table 5.

**Table 5:** Dispersion estimates of the estimators under different temperature measurement scales.

	Celsius ( °C)	Kelvin (K)	Rankine (°Ra)	Fahrenheit (°F)	Romer (°Ro)	Newton (°N)	Reaumur (°*Re*)	Delisle (°D)
*AMM: Kc*	0.9123	0.9123	0.9123	0.9123	0.9123	0.9123	0.9123	0.9123
*AMM: Pprox*	0.8113	0.8113	0.8113	0.8113	0.8113	0.8113	0.8113	0.8113
*AMM: Pdev*	0.1887	0.1887	0.1887	0.1887	0.1887	0.1887	0.1887	0.1887
*AMM: Kcalt1*	0.9123	0.9123	0.9123	0.9123	0.9123	0.9123	0.9123	0.9123
*AMM: Kcalt2*	1.1064	1.1064	1.1064	1.1064	1.1064	1.1064	1.1064	1.1064
*STD*	3.0100	3.0100	5.4180	5.4180	15.8024	99.3295	2.4080	4.5150
*RMM: Kc*	0.9855	0.9987	0.9987	0.9912	0.9862	0.9855	0.9855	1.0155
*RMM: Pprox*	0.9687	0.9972	0.9972	0.9811	0.9702	0.9687	0.9687	0.9675
*RMM: Pdev*	0.0313	0.0028	0.0028	0.0189	0.0298	0.0313	0.0313	0.0325
*RMM: Kcalt1*	0.9855	0.9987	0.9987	0.9912	0.9862	0.9855	0.9855	0.9850
*RMM: Kcalt2*	1.0149	1.0013	1.0013	1.0089	1.0141	1.0149	1.0149	1.0155
*CV*	11.2313	1.0035	1.0035	6.7522	10.6629	11.2313	11.2313	−11.6666

**Keys:***AMM* = Absolute meanic mirroring; *STD* = Standard deviation; *RMM* = Raw meanic mirroring; *CV* = Coefficient of variation.

*Kc* = Kabirian coefficient (of proximity); *Pprox* = Probability of proximity; *Pdev* = Probability of deviation.

*Kcalt1* = Ascending alternate Kabirian coefficient (of proximity); *Kcalt2* = Descending alternate Kabirian coefficient (of proximity).

The outcomes reveal that the standard deviation remains invariant solely to temperature scales transforming a location shift. In contrast, the coefficient of variation and raw meanic deviation demonstrate invariance exclusively to temperature scales transforming a change in scale. Strikingly, the absolute meanic deviation exhibits robust invariance to temperature scales undergoing both a shift in location and a transformation in scale. This resilience underscores its indifference to changes like data representation, whether on a ratio or interval scale.

Similarly, the derivative estimators of the absolute and raw meanic mirroring, including the Kabirian coefficient of meanic proximity, meanic proximity, and the alternate Kabirian coefficient, exhibit a comparable scaloc-invariance and scale-invariance concerning temperature scales. This finding contrasts with the standard deviation and coefficient of variation, emphasizing the nuanced and advantageous properties of the proposed estimators under diverse temperature scale conditions.

### Set duplication-invariance

Table 6 presents a sample of datasets of groups that differs by a variable number of dataset duplications which eventually changes the sample size but the mean remains the same. The impact of set duplication invariance (robustness to dataset duplication) on dispersion estimators was studied.

**Table 6:** Impact of set duplications and sample size on the estimates of dispersion estimators.

Group	Size	Scores	Mean	Population		Sample		Absolute meanic mirroring		Raw meanic mirroring	
				*STD_P_*	*CV_P_*	*STD_S_*	*CV_S_*	*Pprox*	*Pdev*	*Pprox*	*Pdev*
Group 1	4	[2, 9, 23, 25]	14.75	9.6014	65.0944	11.0868	75.1646	0.9252	0.0748	0.8384	0.1616
Group 2	8	[2, 2, 9, 9, 23, 23 25, 25]	14.75	9.6014	65.0944	10.2644	69.5889	0.9252	0.0748	0.8384	0.1616
Group 3	12	[2, 2, 2, 9, 9, 9, 23, 23, 23, 25, 25, 25]	14.75	9.6014	65.0944	10.0284	67.9889	0.9252	0.0748	0.8384	0.1616
Group 4	16	[2, 2, 2, 2, 9, 9, 9, 9, 23, 23, 23, 23, 25, 25, 25, 25]	14.75	9.6014	65.0944	9.9163	67.2293	0.9252	0.0748	0.8384	0.1616
Group 5	28	[2, 2, 2, 2, 2, 2, 2, 9, 9, 9, 9, 9, 9, 9, 23, 23, 23, 23, 23, 23, 23, 25, 25, 25, 25, 25, 25, 25]	14.75	9.6014	65.0944	9.7776	66.2889	0.9252	0.0748	0.8384	0.1616
Group 6	200	[2, 9, 23, 25] * 50	14.75	9.6014	65.0944	9.6255	65.2578	0.9252	0.0748	0.8384	0.1616
Group 7	4000	[2, 9, 23, 25] * 1000	14.75	9.6014	65.0944	9.6135	65.1760	0.9252	0.0748	0.8384	0.1616
Group 8	4 ×*n*	[2, 9, 23, 25] * n, n∈N	14.75	9.6014	65.0944	9.6014	65.0944	0.9252	0.0748	0.8384	0.1616

**Keys:***STD_P_* = Population standard deviation; *CV_P_* = Population coefficient of variation; *STD*_S_ = Sample standard deviation.

*CV_S_* = Sample coefficient of variation; *Pprox* = Probability of proximity; *Pdev* = Probability of deviation.

The outcomes reveal that despite having an identical central tendency and score distribution, all the classical estimators, the proposed statistical absolute, and raw meanic mirroring are robust to set duplication(s). However, these classical estimators lack the robustness to set duplication(s) only for their sample estimates (i.e., sample standard deviation and coefficient of variation).

## Discussion

### Transformation invariance

The investigation into dispersion estimates under normal distribution using classical and proposed methods unveiled notable invariance characteristics. Categorizing estimators into location-variance, scale-invariance, or scaloc-invariance, the study revealed the standard deviation's location invariance and the coefficient of variation/raw meanic deviation's scale invariance. The absolute meanic deviation uniquely emerged as scaloc-invariant, resisting both location shift and scaling effects. Despite increased sample sizes, each estimator maintained an invariance, converging consistently toward accurate population estimates. The unique scaloc-invariant estimator addresses challenges in interpreting dispersion estimates in research outcomes. Darling [19] and Everitt et al. [20] highlighted result interpretation as a perplexing aspect of data analysis. This innovation ensures the interpretation of results is independent of the mean, enabling reliable comparisons of two or more disparate groups and characteristics. However, where a location shift transformation of a variable is a specific statistical concern for a location shift-invariant estimator, statistical mirroring is not a suitable alternative to standard deviation, hence the standard deviation remains an efficient tool.

However, the coefficient of variation displayed sensitivity to mean direction, introducing variability in dispersion estimates. In contrast, proposed estimators demonstrated stability, independent of mean direction. Derivative estimators of the proposed method (i.e., Kabirian coefficient of meanic proximity and meanic proximity) exhibited comparative scaloc and scale invariance, contrasting with standard deviation and coefficient of variation behaviors.

Moreover, standard deviation, though location-invariant, lacked bounds, posing challenges in interpretation. Bounded proposed estimators, including absolute and raw meanic deviations, offered clear and predictable ranges, enhancing robustness. Derivative estimators like meanic proximity provided additional advantages with a unified and well-defined probabilistic boundary. These findings emphasize the stability and practical applicability of proposed estimators in diverse scenarios, advancing data analysis and interpretation methodologies. The findings also confirmed the conceptual and theoretical uniqueness of the Kabirian-based optinalysis, which is the inspiring backbone of proposing this methodology of statistical mirroring [9].

### Efficiency and relative efficiency

The systematic evaluation of dispersion estimates under normal distribution highlighted the efficiency and relative efficiency of both classical and proposed estimators. Employing simulation parameters with diverse sample sizes and transformations, the assessment, as depicted in Fig. 4, revealed commendable efficiencies across estimators. This reliability persisted even with subtle variations as sample sizes increased.

Efficiency, a critical property of estimators [11,[21], [22], [23], was notably commendable across all methods. The standard deviation exhibited slightly higher efficiency than the absolute meanic deviation with statistical significance (*P* < 0.05), and the coefficient of variation was asymptotically equal to the raw meanic deviation. Derivative estimators, including the Kabirian coefficient of meanic proximity, meanic proximity, and alternate Kabirian coefficient, demonstrated comparable efficiency and precision in handling random samples from a symmetrical normal distribution. The standard deviation demonstrated superior efficiency, benefitting from the theoretical foundation upon which the data generator is constructed. Hence, engaging in self-versus-non-self comparisons becomes inherently challenging.

Efficiency is a crucial attribute in estimator evaluation, ensuring precision and reliability in data analysis [11,[21], [22], [23]. The observed trade-offs between different estimators highlight the nuanced balance required for practical applications, emphasizing the significance of these efficiency characteristics in enhancing the reliability of results in diverse scenarios.

### Sensitivity to location shift contaminations

The assessment of sensitivity to location shift contaminations in dispersion estimates, involving both classical and proposed estimators, revealed noteworthy findings. The absolute meanic deviation demonstrated superior and heightened resistance, maintaining a below 5% relative error against a 7.90% contamination level. Its lower maximum sensitivity effect, forming a symmetrical curve around the 50% contamination level, signifies robustness. In contrast, the standard deviation exhibited limited resistance, achieving below 5% relative error only at a 1.50% contamination level, with a higher maximum sensitivity effect.

Similarly, the raw meanic deviation showcased robust resistance, maintaining below 5% relative error against a 2.20% contamination level, with a lower maximum sensitivity effect. Conversely, the coefficient of variation displayed limited resistance, maintaining below 5% relative error only at a 1.50% contamination level, with an equivalent lower maximum sensitivity effect.

The proposed estimators exhibited superior sensitivity owing to their methodological approach, characterized by an independent isoreflective mapping of each data point (whether raw data or its absolute distance from the center) to its mean value (the principal value). This independent mapping ensures that outliers do not exert a disproportionately significant effect on the estimate. In contrast, the standard deviation and subsequently the coefficient of variation relies on the average of squared distances from the mean, leading outliers to exert more weight on the estimates, introducing a substantial bias. Despite mathematical advantages, squaring the distances of data points from the mean increases the sensitivity to outliers.

Derivative estimators, including the Kabirian coefficient of meanic proximity, meanic proximity, and the alternate Kabirian coefficient, demonstrated heightened robustness and resilience to location shift contaminations when compared to the standard deviation and coefficient of variation. This emphasis on robustness aligns with the importance of accurate and reliable data analysis, especially in scenarios involving contaminations [3,^8^,^24^,25]. The novel aspect lies in the comprehensive evaluation of multiple estimators and their distinct responses to location shift contaminations, shedding light on their suitability for robust data analysis. The findings also reaffirmed the conceptual and theoretical robustness of the Kabirian-based optinalysis, which is the exciting backbone of proposing this methodology of statistical mirroring [9].

### Sensitivity to scaling contaminations

The examination of sensitivity to scaling contaminations in dispersion estimates, encompassing both classical and proposed estimators, yielded notable insights. The absolute meanic deviation showcased superior and heightened resistance, maintaining a below 5% relative error against a 12.40% scaling contamination, with a lower maximum sensitivity effect. In contrast, the standard deviation displayed limited resistance, achieving below 5% relative error only at a 3.50% scaling contamination level, with a higher maximum sensitivity effect growing polynomially.

Similarly, the raw meanic deviation demonstrated robust resistance, maintaining below 5% relative error against a 4.30% scaling contamination, with a lower maximum sensitivity effect growing polynomially. Conversely, the coefficient of variation exhibited limited resistance, maintaining below 5% relative error only at a 3.50% scaling contamination level, with a higher maximum sensitivity effect growing polynomially.

The methodological approach of the proposed estimators demonstrated heightened sensitivity by employing an independent isoreflective mapping for each data point to its mean value. This unique mapping ensures that outliers have a limited impact on the estimate. Conversely, the standard deviation relies on averaging squared distances from the mean, allowing outliers to exert more influence on estimates and introducing bias.

Derivative estimators, including the Kabirian coefficient of meanic proximity, meanic proximity, and the alternate Kabirian coefficient, exhibited more comparative robustness and resilience to scaling contaminations compared to the standard deviation and coefficient of variation. This emphasis on resistance to scaling contaminations contributes to the robustness of estimators, a crucial property for ensuring accuracy and reliability in data analysis [3,^8^,^24^,25]. The novelty lies in the comprehensive evaluation of multiple estimators and their distinct responses to scaling contaminations, providing valuable insights into their suitability for practical applications. The findings also reaffirmed the conceptual and theoretical robustness of the Kabirian-based optinalysis, which is the exciting backbone of proposing this methodology of statistical mirroring [9].

### Dispersion under temperature measurement scales

The examination of dispersion estimates under various temperature scales using classical and proposed estimators reveals robust invariance patterns. The standard deviation exhibits invariance solely to temperature measurement scales undergoing a location shift, while the coefficient of variation and raw meanic deviation are invariant exclusively to temperature measurement scales changing unit scale. Notably, the absolute meanic deviation demonstrates robust invariance to temperature measurement scales undergoing both location shift and unit scale transformation, emphasizing its adaptability to different data representations.

Derivative estimators, including the Kabirian coefficient of meanic proximity, meanic proximity, and the alternate Kabirian coefficient, display comparable scaloc-invariance and scale-invariance across temperature scales. This contrasts with the standard deviation and coefficient of variation, highlighting the nuanced and advantageous properties of proposed estimators in ensuring comparability, accuracy, and reliability of results in diverse temperature scale scenarios. The novelty lies in uncovering the distinctive invariance characteristics of various estimators under real-life temperature measurements, contributing to their applicability in data analysis across different scales. The findings justified the conceptual and theoretical uniqueness and robustness of the Kabirian-based optinalysis, which is the exciting backbone of proposing this methodology of statistical mirroring [9].

### Set duplication-invariance

In research, employing the sample standard deviation as an alternative to the population standard deviation is common when dealing with a subset of the population [26]. Accessing data for the entire population is often challenging, making the use of sample estimates of dispersion statistically essential. However, the sample estimate of standard deviation and coefficient of variation is affected by repeated or duplicate observations, artificially inflating the sample size [27].

In sociology and economics, using the coefficient of variation to estimate demographic diversity from a sample introduces challenges when comparing groups with differing sample sizes or dealing with repeated observations [28]. When two or more groups exhibit likely duplicate or repeated outcomes, variations emerge, even though they should theoretically share the same variability. A robust solution to this issue lies in the application of statistical meanic mirroring, which remains unaffected by dataset duplications, or employing a corrected version of the coefficient of variation introduced by Smithson [29]. Both approaches ensure comparability across diverse sample sizes.

### Methodological advances of statistical meanic mirroring

Table 7 outlined some desirable characteristics of estimators to compare the methodological advances of statistical meanic mirroring. These methodological advances provide a comprehensive overview of the unique characteristics and capabilities of each statistical method, aiding researchers and analysts in selecting the most suitable approach for their specific data analysis needs.

**Table 7:** Comparative methodological features of statistical dispersion estimators.

Characteristics/features	*AMM*	*STD*	*RMM*	*CV*
a. Takes into consideration all data points of the distribution	Yes	Yes	Yes	Yes
b. Applicable to measurements on both interval scale and ratio scale	Yes	No	Yes	No^1^
c. Probabilistic bounded range of estimates	Yes	No	Yes	Yes^1^
d. Produce negative estimates	No	No	Yes^2^	Yes^3^
e. Applicable to variables containing both negative and positive values	Yes	Yes	Yes	Yes
f. Location-invariance (invariance to location shift transformation)	Yes	Yes	No	No
g. Scale-invariance (invariance to scaling transformation)	Yes	No	Yes	Yes
h. Scaloc-invariance (invariance to both location shift and scaling transformations)	Yes	No	No	No
i. Set duplication-invariance (invariance to multiple duplications of a set)	Yes	Yes^4^	Yes	Yes^5^
j. Insensitive to mean value close to or approaching zero	Yes	Yes	No	No
k. Insensitive to variables whose mean value can be zero	Yes	Yes	No	No
l. Heightened resistance to a small set of outliers	Yes	No	No	No

**Keys:***AMM* = Absolute meanic mirroring; *STD* = Standard deviation; *RMM* = Raw meanic mirroring; *CV* = Coefficient of variation.

**Notes:** No^1^ = Is meaningless for an interval scale.

Yes^1^ = The boundary is lost when the mean is greater than the standard deviation.

Yes^2^ = Negativity is due to a resultant weight of integers between the dataset and its mirror set.

Yes^3^ = Negativity is due to the directionality of a mean.

Yes^4^ = The set duplication-invariance is exclusive to population *STD*.

Yes^5^ = The set duplication-invariance is exclusive to *CV* derived from a population *STD*.

## Conclusion

In conclusion, this study delved into the comprehensive evaluation of dispersion estimates, focusing on transformation invariance, efficiency, and sensitivity to contaminations. The primary objective was to propose new robust alternative estimators of statistical dispersion and provide a nuanced understanding of the behavior and properties of classical and proposed estimation methods. The findings shed light on critical aspects that significantly contribute to the advancement of statistical methodologies. Because of the methodological broadness in types of statistical mirroring, the limitation on the applicability and evaluation is restricted to only one of the approaches, the statistical meanic mirroring. For comparison, standard deviation and coefficient of variation were chosen as the reference classical estimator of statistical dispersion.

Statistical dispersion involves preprocessing transformations, statistical mirror design, and optimization to transmute a univariate set into a bivariate one, facilitating the fitting of an isomorphic optinalysis model. This proposed methodology extends beyond deviations around the mean or median. Statistical mirroring introduces alternative estimators that account for proximity around any center, including the mode, maximum, and minimum, within a distribution. This broadens the scope of statistical dispersion estimations, opening the door to diverse applications across various research fields.

The investigation into transformation invariance uncovered distinctive characteristics of each estimator. Notably, the proposed absolute meanic deviation emerged as scaloc-invariant, showcasing robustness against both location shift and scaling effects. This property is particularly novel and significant, as it enhances the estimator's applicability across diverse scenarios, irrespective of changes in data representation.

Efficiency and relative efficiency assessments revealed commendable performance across all estimators, with statistically significant differences observed only between standard deviation and absolute meanic mirroring. The proposed estimators demonstrated competitive efficiency compared to traditional measures, presenting a balance between precision and practicality. These findings underscore the reliability and precision of the proposed estimators, providing researchers with valuable tools for data analysis and interpretation.

Sensitivity to contaminations, both in terms of location shift and scaling, further highlighted the robustness of the proposed estimators. The absolute and raw meanic deviations exhibited lower sensitivity to contaminations compared to standard deviation and coefficient of variation, emphasizing its resilience in maintaining accurate estimates even under adverse conditions.

Moreover, the exploration of invariance under different temperature scales revealed the proposed absolute meanic deviation as uniquely scaloc-invariant, showcasing its versatility and suitability for diverse data representations. The derivative estimators also exhibited advantageous properties in this context, reinforcing the novelty and applicability of the proposed methods.

Set duplication-invariance is crucial for unbiased estimates in subsets with repeated data. Traditional sample standard deviation faces challenges in accuracy due to duplications. The coefficient of variation, used for demographic diversity, struggles with variable sample sizes and repeated observations. The robust statistical meanic mirroring ensures reliable comparisons across diverse sample sizes. These innovations underscore the importance of set duplication-invariance, offering advanced methodologies for consistent and unbiased estimations in research applications.

Generally, the investigation into transformation invariance revealed the novel scaloc-invariant property of the proposed absolute meanic deviation, enhancing its applicability across diverse scenarios. Efficiency assessments demonstrated competitive performance, balancing precision and practicality, providing reliable tools for data analysis. Sensitivity tests highlighted the robustness of proposed estimators, especially the absolute meanic deviation, under adverse conditions. Invariance exploration under different temperature scales showcased versatility. Set duplication-invariance, essential for unbiased estimates, was addressed by the robust statistical meanic mirroring, emphasizing advanced methodologies for consistent and unbiased estimations in research applications.

## Recommendations

For future research, it is recommended to:

1. Expand sample variability: Extend research to encompass a broader spectrum of data distributions (such as gamma, beta, Poisson, exponential, log-normal, uniform, chi-squared, etc.), ensuring a comprehensive understanding of how the proposed estimators perform under various statistical conditions.
2. Compare with alternative methods: Extend comparative studies to include a broader range of existing statistical methods, providing a more comprehensive understanding of the strengths and weaknesses of the proposed estimators.
3. Address multivariate settings: Explore the behavior of the proposed estimators in multivariate settings, assessing their performance and invariance properties in more complex data structures.
4. Consider non-normal distributions: Investigate the robustness and efficiency of the proposed estimators in the context of non-normal distributions, expanding their applicability to a wider array of data types.
5. Validate with real data: More validation of the findings using real-world datasets to ensure the practical relevance and generalizability of the proposed estimators in applied statistical analyses.
6. Address data quality issues: Assess the performance of the proposed estimators in the presence of data quality issues, such as missing data or measurement errors, to ensure their robustness in less-than-ideal conditions.

These recommendations aim to guide future research endeavors, ensuring a deeper understanding of the proposed estimators' capabilities, limitations, and potential advancements in statistical methodology.

## Ethics statements

The author declares to comply with the Journal's ethical guidelines.

## CRediT author statement

No other authors state their contribution.

## Declaration of generative AI and AI-assisted technologies in the writing process

During the preparation of this work, the author used *ChatGPT* and *Grammarly* to improve language and readability. After using this tool/service, the author reviewed and edited the content as needed and took full responsibility for the content of the publication.

## Funding

This research did not receive any specific grant from funding agencies in the public, commercial, or not-for-profit sectors.

## Declaration of competing interest

The authors declare that they have no known competing financial interests or personal relationships that could have appeared to influence the work reported in this paper.

## Data Availability

Python codes/scripts and computer application for statistical mirroring, and other processes of simulating, analyzing, and evaluating dispersion estimators Python Scripts for Simulating, Analyzing, and Evaluating Dispersion Estimators (Original data) (Mendeley Data) A Python Code for Statistical Mirroring (Original data) (Mendeley Data) Statistical Mirroring Computer Application for Robust Dispersion Estimation (Original data) (Mendeley Data)

## Appendix A: Plots at lower levels of location shift contamination

The sensitivity to contamination of dispersion estimates from both classical and proposed estimators under Gaussian mixture model distributions was scrutinized using the simulation parameters outlined in Fig. 2. The line graph of the sensitivity evaluation is shown in Figs. A1–A2. This plot presented the behavior of the estimates of the estimators under low levels of location shift contaminations to allow some predictions and evaluation.

## Appendix B: Plots at lower levels of scaling contamination

The sensitivity to contamination of dispersion estimates from both classical and proposed estimators under Gaussian mixture model distributions was scrutinized using the simulation parameters outlined in Fig. 2. The line graph of the sensitivity evaluation is shown in Figs. B1–B2. This plot presented the behavior of the estimates of the estimators under low levels of scaling contaminations to allow some predictions and evaluation.
